# Flexible fluorine-thiol displacement stapled peptides with enhanced membrane penetration for the estrogen receptor/coactivator interaction

**DOI:** 10.1016/j.jbc.2024.107991

**Published:** 2024-11-13

**Authors:** Robert Maloney, Samuel L. Junod, Kyla M. Hagen, Todd Lewis, Changfeng Cheng, Femil J. Shajan, Mi Zhao, Terry W. Moore, Thu H. Truong, Weidong Yang, Rongsheng E. Wang

**Affiliations:** 1 Department of Chemistry, Temple University, Philadelphia, Pennsylvania, USA; 2 Department of Biology, Temple University, Philadelphia, Pennsylvania, USA; 3 Department of Biochemistry, Molecular Biology and Biophysics, University of Minnesota, Minneapolis, Minnesota, USA; 4 Masonic Cancer Center, University of Minnesota, Minneapolis, Minnesota, USA; 5 Department of Pharmaceutical Sciences, College of Pharmacy, University of Illinois at Chicago, Chicago, Illinois, USA

**Keywords:** fluorine, stapled peptides, membrane, nucleus uptake, estrogen receptor, protein protein interaction(s), fluorine displacement reaction (FDR)

## Abstract

Understanding how natural and engineered peptides enter cells would facilitate the elucidation of biochemical mechanisms underlying cell biology and is pivotal for developing effective intracellular targeting strategies. In this study, we demonstrate that our peptide stapling technique, fluorine-thiol displacement reaction (FTDR), can produce flexibly constrained peptides with significantly improved cellular uptake, particularly into the nucleus. This platform confers enhanced flexibility, which is further amplified by the inclusion of a D-amino acid, while maintaining environment-dependent α helicity, resulting in highly permeable peptides without the need for additional cell-penetrating motifs. Targeting the estrogen receptor α (ERα)-coactivator interaction prevalent in estrogen receptor-positive (ER+) breast cancers, we showcased that FTDR-stapled peptides, notably SRC2-LD, achieved superior internalization, including cytoplasmic and enriched nuclear uptake, compared to peptides stapled by ring-closing metathesis. These FTDR-stapled peptides use different mechanisms of cellular uptake, including energy-dependent transport such as actin-mediated endocytosis and macropinocytosis. As a result, FTDR peptides exhibit enhanced antiproliferative effects despite their slightly decreased target affinity. Our findings challenge existing perceptions of cell permeability, emphasizing the possibly incomplete understanding of the structural determinants vital for cellular uptake of peptide-like macromolecules. Notably, while α helicity and lipophilicity are positive indicators, they alone are insufficient to determine high-cell permeability, as evidenced by our less helical, more flexible, and less lipophilic FTDR-stapled peptides.

Small-molecule-based endocrine therapies are the most widely used treatment option for estrogen-receptor positive (ER+) breast cancer (BC) and are almost always used as the first treatment course. These hormonal therapies are split into two groups based on their mechanism of action, selective estrogen receptor modulators and selective estrogen receptor degraders. Selective estrogen receptor modulators (*e.g.*, tamoxifen) modulate estrogen receptor activity by blocking coactivator recruitment leading to reduced gene transcription (1). Selective estrogen receptor degraders (*e.g.*, fulvestrant) tightly bind to the ER and inhibit its dimerization which prevents it from reaching the nucleus (trapping it in the cytoplasm) where it is subsequently degraded (1). Previous reports estimate that nearly 40% of ER + BC patients will gain resistance to these endocrine therapies (1). While a range of treatments is available, ER + BC remains highly lethal and has a notable recurrence rate (1, 2). Furthermore, extending the duration of endocrine therapy does not reduce the risk of recurrence and has been linked to mutations in the ER-encoded gene (*ESR1)* fueling drug resistance (1, 3). The double-edged sword of small-molecule drugs’ therapeutic efficacy and increased drug resistance underscores the urgent need for new therapies that can appropriately target the large interface between estrogen receptor α (ERα) and steroid receptor coactivator 2 (SRC2).

The development of bioactive peptides is rapidly gaining traction in the field to probe intracellular protein targets. However, the use of peptides as intracellular probes is hindered by their intrinsic limitations of proteolytic stability, affinity, and membrane permeability (4, 5, 6). A prevalent method to combat these shortcomings is by way of chemical modification, covalently crosslinking amino acid side chains to provide a rigid support to the peptide backbone thereby promoting the formation of an α-helix independent of neighboring groups within the context of proteins. To date, the most widely used and foundational method is ring-closing metathesis (RCM) peptide stapling, wherein two unnatural amino acids (S_5_ or R_8_) are incorporated into a peptide chain at positions (*i, i +* 4) or *(i, i +* 7) during solid-phase synthesis and can be readily crosslinked to form a hydrocarbon staple, reinforcing the helical backbone (1). Peptide stapling results in vastly increased α-helical secondary structure, proteolytic and thermal stability, and improved cellular uptake relative to unmodified linear peptides (7, 8). The rigidity of the crosslinker constrains a peptide into a more biologically active helical conformation (9, 10, 11). There have been a myriad of reports demonstrating increased binding affinity of stapled over linear peptides, highlighting their increased interest for use as therapeutics (12). Recently, a RCM-stapled p53-mimetic peptide has entered clinical trials as a promising cell-penetrating anticancer drug (13). However, the RCM stapling platform has some drawbacks. For instance, the added hydrophobicity of the crosslinker is associated with increased hemolytic activity (14, 15, 16). Hemolytic properties are often desirable for antibacterial purposes, but they cause undesirable cytotoxicity in mammalian cells (16). Most important of all, RCM-stapled constructs often still exhibit poor to mild cell penetrability (4, 17, 18).

To address this, one common solution is to conjugate a peptide with a cell-penetrating motif, such as TAT or polyarginine, to facilitate cellular uptake (19, 20). Yet, this bears the cost of tethering excessive positively charged or hydrophobic groups which can detract from the native peptide’s secondary structure and function, target affinity, and potentially compromise the integrity of cell membranes (21, 22, 23). In response to this, we have designed a new class of stapled peptides, achieved through facile peptide synthesis incorporating the unnatural amino acid X_L/D_, containing a fluoroacetamide side-chain (Fig. S1) (24, 25). Fluorine-thiol displacement reaction (FTDR) allows for the selective crosslinking of a benzene-dithiol “staple” in unprotected peptides representing a unique stapling scaffold, capable of generating peptides with markedly enhanced plasma membrane permeability, reinforced α-helicity, and metabolic stability (24, 25). We have previously shown the trend that peptides stapled through FTDR in either X_L_/X_D_ or X_L_/X_L_ combination for *i* or *i* + 4 sites display similarly enhanced cellular uptake compared to those stapled by RCM (25).

Here, we present our latest findings on the peptide derivatives, SRC2-LD and SRC2-LL, designed from the interaction between ERα and SRC2, which is a well-established target for ER + BC (10, 18). Phillips *et al.* initially investigated the parent SRC2-derived peptide sequence, HKILHRLLQDS, and evaluated different stapled peptide analogues as potential estrogen receptor inhibitors (10). It was discovered that the replacement of two key hydrophobic residues on the binding face of the SRC2-ER interaction, isoleucine and leucine, with an olefin staple led to an increase in receptor-binding affinity and peptide helicity (10). Upon activation by estradiol (E2), ERα adopts an active conformation necessary to bind SRC2 and drive transcription and cancer cell growth (26). The binding face of the coactivator interaction is composed of a short, conserved α-helical sequence making the use of stapled peptide mimics a logical inhibition strategy, as demonstrated previously (10, 18). Since ERα is a nuclear receptor, any potential inhibitor would need to be, at the very least, capable of crossing the plasma membrane and ideally the nuclear membrane to be effective. The application of RCM stapling to SRC2 mimetics (SRC2-RCM) has previously encountered challenges related to membrane permeability, thereby limiting the related inhibitor designs to TAT-like or poly-arginine conjugates (18). Excitingly, our FTDR stapled SRC2 peptides, particularly the one with X_D_ at the *i* + 4 site (SRC2-LD), were demonstrated to possess enhanced penetration toward not only the plasma membrane but also the nuclear membrane, which translated toward their enhanced growth inhibition of the ER + BC cell line MCF-7.

## Results

### FTDR and RCM stapling of SRC2 peptide analogues

**Figure 1:**
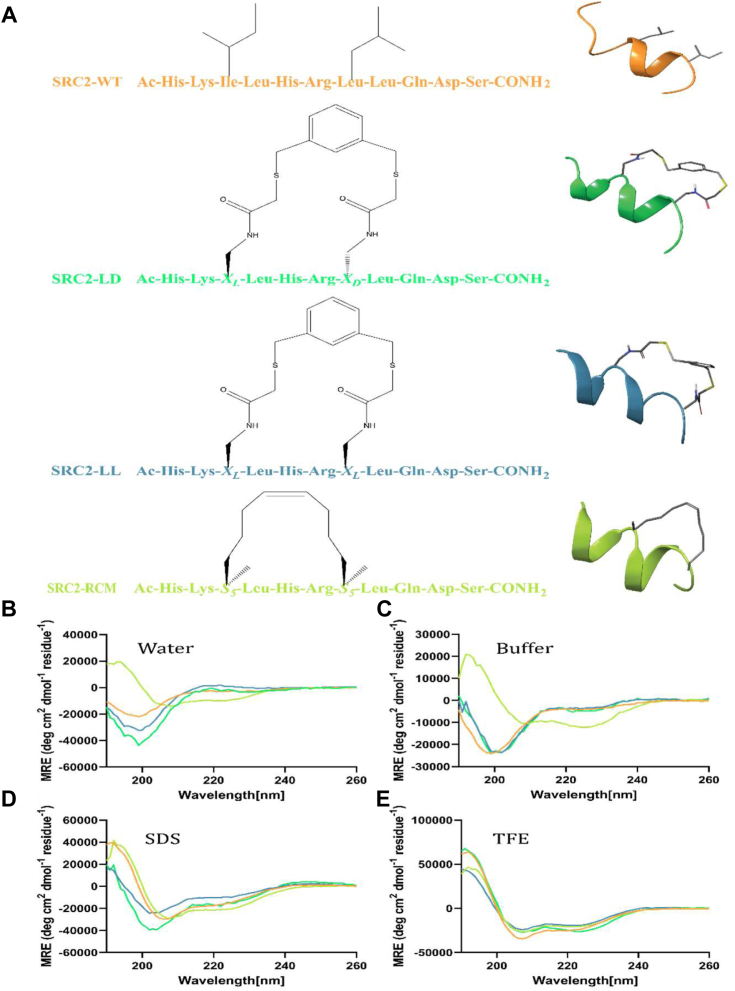
**The stapled SRC2 peptides and their secondary structure analysis.***A*, schematic representation of peptide sequences used in this study, highlighting staple positions and chemical compositions for each variant. SRC2-WT and SRC2-RCM are derived from their respective crystal structures (PDB IDs: 7NEL and 5DXE) (27, 66). SRC2-LL and SRC2-LD structures were generated from the SRC2-RCM base structure with the replacement of FTDR-specific amino acids (X_L_ and X_D_) and the corresponding staple. All cartoon peptides were visualized using Schrödinger Suite software (https://www.schrodinger.com). *B*–*E*, circular dichroism (CD) spectra demonstrating secondary structure characteristics of peptide derivatives in different solvents: (*B*) water, (*C*) phosphate buffer (45 mM, pH 7.4), (*D*) SDS (30 mM, pH 7.4), and (*E*) 50% TFE in water, pH 7.4. The CD spectra colors correspond to the peptide variants as indicated in panel (*A*). FTDR, fluorine-thiol displacement reaction; PDB, Protein Data Bank; RCM, ring-closing metathesis; SRC2, steroid receptor coactivator 2; TFE, 2,2,2-trifluoroethanol.

We selected the peptide sequence HKILHRLLQDS as the basis for our study, drawing inspiration from existing literature (10, 18, 27). FTDR stapling was performed as done previously (25), using preactivated 1,3-benzenedimethanethiol to form a covalent linkage between X_L_ and X_L/D_ (Fig. 1*A*). Reaction progress was monitored by LCMS, observing > 90% completion after 16 h. The stapling yield after HPLC purification was approximately 63% and 60% for SRC2-LL and SRC2-LD, respectively. RCM stapling was performed on-beads (28), using Grubb’s first-generation catalyst for a total reaction time of 4 h. After peptide cleavage and subsequent HPLC purification, SRC2-RCM yield was determined to be ∼ 69%. All these final stapled peptides along with the WT were analyzed by LC-MS post HPLC purification confirming a purity of > 95% (Table S1, Figs. S23–S20).

### Flexibility in the folding of FTDR-stapled SRC2 peptides

We have shown previously that FTDR stapled peptides are highly tolerant of D-amino acid incorporation in the N-terminal (*i + 4)* staple position (25), but herein, SRC2-LD is unique in that, it is both more helical and induced more environment-dependent folding than its LL counterpart. The incorporated D-amino acid also offers an innate advantage over pure L-amino acid-containing biomolecules, as the inclusion of even a single D-amino acid has been reported to greatly enhance proteolytic resistance (29, 30, 31, 32). We evaluated the secondary structures of all SRC2 peptides and observed a general trend: polar solvents (water and phosphate buffer) resulted in weaker helicities while membrane-mimicking solvents (2,2,2-trifluoroethanol [TFE] and SDS micellar solution) greatly enhanced helicity, especially for the FTDR-stapled peptides (Fig. 1, *A* and *B*, Table 1). SRC2-RCM exhibited approximately 2 to 3 times greater helicity than the other peptides in aqueous solvents, and its helicity nearly doubled in the helix-stabilizing solvents, SDS and TFE (Table 1). Similar to the WT peptide, SRC2-WT (17.0% α helicity in phosphate buffer), our FTDR-stapled peptides display mild intrinsic helicity in the aqueous solutions (15.4% for SRC2-LL and 21.0% for SRC2-LD in phosphate buffer) which indicates that our staple is less constraining and confers high innate flexibility.

**Table 1:** Percent of α helicity calculated based on 222 nm MRE values from the CD spectra in water, phosphate buffer, SDS-micellar solution, and TFE

Peptide	Solvent	α-helicity (%)
SRC2-WT	Water	12.8
	Buffer	17
	SDS	57.9
	TFE	84
SRC2-LD	Water	12
	Buffer	21
	SDS	63
	TFE	89.1
SRC2-LL	Water	n.d.
	Buffer	15.4
	SDS	37.2
	TFE	67.6
SRC2-RCM	Water	36.2
	Buffer	42
	SDS	74.1
	TFE	70.9

Interestingly, although SRC2-LL lacks a defined secondary structure in water (n.d., Table 1), in nonaqueous solutions it adopts a much more helical structure (37.2% in SDS solution and 67.6% in TFE), approaching that of RCM (74.1% in SDS and 70.9% in TFE). Notably, SRC2-LD is universally more α-helical than SRC2-LL in all the solutions evaluated, and it also displays significant α-helical variations across different solvents, exhibiting the maximal helicity in the least polar solvent (89.1% in TFE) out of the group. Statistical analysis of the calculated percentage of α-helicity demonstrates significant differences between these four groups (Fig. S2).

The trend of increasing helicity when moving from hydrophilic to hydrophobic environments mirrors that observed in the WT sequence, demonstrating a flexibility comparable to that of the native, unstapled peptide. This intrinsic flexibility conferred by FTDR stapling likely enables peptides to adopt a diverse set of conformations depending on the microenvironment in biological systems.

### *In silico* structural analysis of SRC2 peptide analogues

In order to investigate the possible conformations underlying the environment-dependent α-helicity observed above, we performed *in silico* computational modeling in water and chloroform. As shown in Table 2, common permeability-related factors including LogP, polar surface area (PSA), radius of gyration (R_gyr_), number of hydrogen bond donors/acceptors (nHBD/nHBA), and number of intramolecular hydrogen bonds (nIMHB) were calculated. Compared to the SRC2 peptide stapled by RCM, FTDR-stapled peptides deviate from established guidelines for permeable molecules (33, 34, 35, 36), possessing lower LogP values and higher values of PSA, R_gyr_, and nHBD/nHBA (Table 2).

**Table 2:** Conformational sampling of cell permeability-related parameters as presented in average values

Solvent peptide	Molecular weight (Da)	nHBD/nHBA^a^	LogP^b^	Rgyr^c^	PSA^d^	nIMHB^e^	Molecular volume^f^
Water							
LD	1595.7	23/20	−8.82	6.43	690.1	14.0	3839.65
LL	1595.7	23/20	−8.76	6.71	697.1	14.0	3915.01
RCM	1423.8	21/16	−7.85	6.14	618.3	11.7	3607.95
CHCl_3_							
LD	1595.7	23/20	−7.74	6.02	604.8	19.5	3696.14
LL	1595.7	23/20	−8.04	6.51	646.1	18.3	3850.35
RCM	1423.8	21/16	−7.13	5.92	570.5	16.6	3512.97

Total number of conformations in water = 1404 for LD, 1266 for LL, and 706 for RCM. Total number of conformations in chloroform = 567 for LD, 393 for LL, and 608 for RCM.

a Number of hydrogen-bond donors (HBD)/hydrogen bond acceptors (HBA).

b Average LogP values.

c Average radius of gyration.

d Average 3D polar surface area (PSA).

e Average number of intramolecular hydrogen bonds (IMHB).

f Average molecular volume (Å^3^).

**Figure 2:**
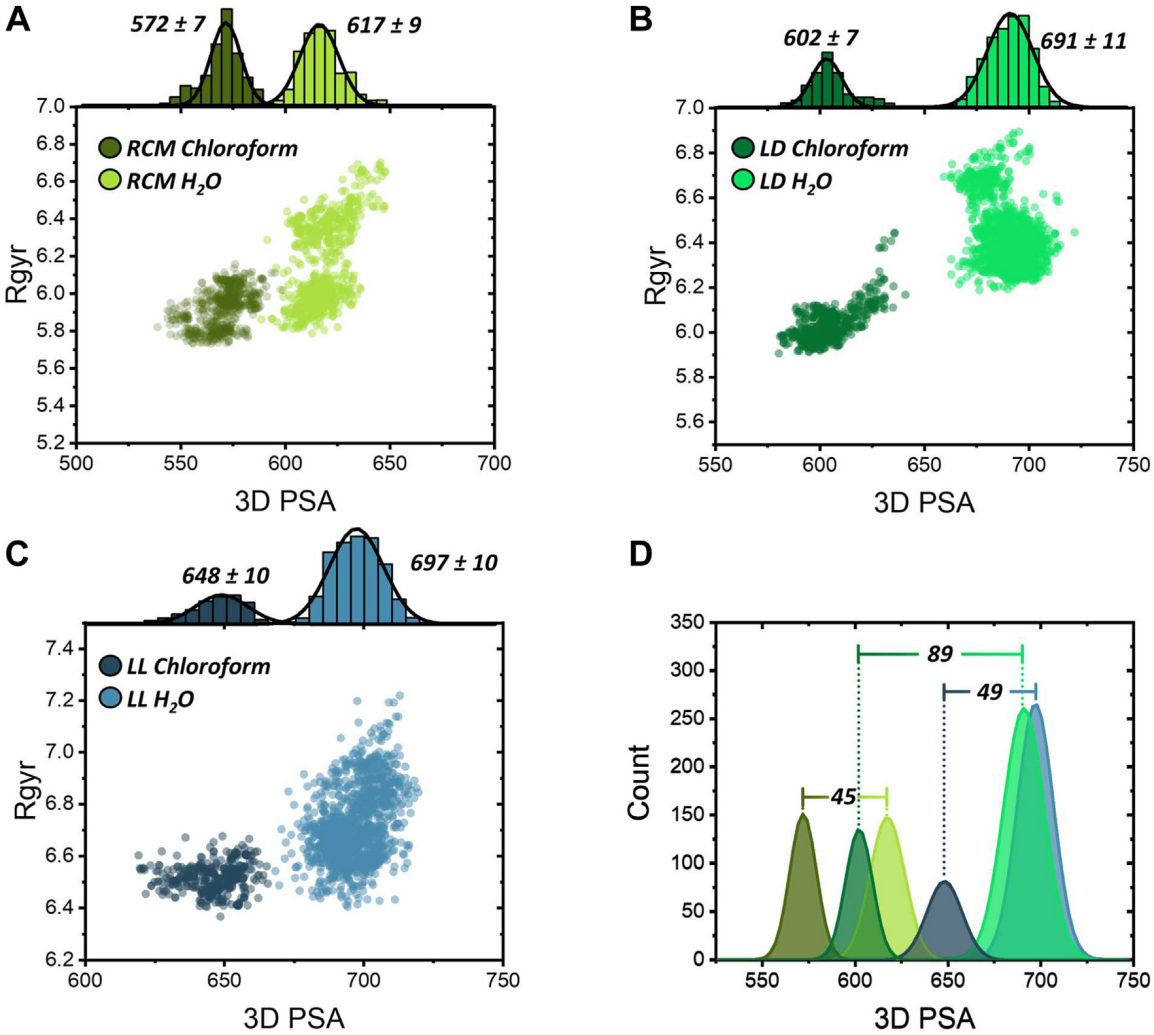
**Plots of radius of gyration (Rgyr) *versus* 3D polar surface area (PSA) of stapled peptides.** (*A*) SRC2-RCM, (*B*) SRC2-LD, and (*C*) SRC2-LL modeled in water and chloroform. Structures were produced in Maestro using both Mixed-torsional/Low-mode (LMOD) and Monte-Carlo torsional conformational sampling. Histogram plots fitted with a Gaussian function ± 1 SD show average PSA values of each peptide in water/chloroform. *D*, histogram plot of all peptide groups LD (*green*), LL (*blue*), and RCM (*yellow*). Inset values represent the difference in the mean PSA values between water and chloroform for each peptide group, illustrating the innate flexibility of SRC2-LD. RCM, ring-closing metathesis; SRC2, steroid receptor coactivator 2.

Based on 2D-plots of R_gyr_ *versus* 3D-PSA, R_gyr_ *versus* LogP, and IMHB *versus* 3D-PSA (Fig. 2 and Figs. S3–S9), SRC2-RCM displayed mild variability in predicted conformations between water/chloroform, favoring more compact, nonpolar structures (Figs. 2*A* and S3). On the contrary, *in silico* modeling of SRC2-LD exhibited the widest variety of predicted conformations in both the solvents (Figs. 2*B* and S4), with R_gyr_ and 3D-PSA values in chloroform approaching those of RCM (6.02 and 604.8 *versus* 5.92 and 570.5, for R_gyr_ and PSA, respectively, Table 2). In terms of absolute values, SRC2-LL exhibited the highest R_gyr_ and PSA in both water and chloroform, indicating its general preference for looser, more polar conformations (Figs. 2*C* and S5). Combining the pool of all conformations of the three stapled peptide groups illustrates the innate flexibility of the FTDR-stapled peptides, especially SRC2-LD (Figs. S6–S9). It is worth noting that the difference in 3D-PSA of modeled conformations between water and chloroform for SRC2-LD (∼89) is nearly double compared to both RCM (∼45) and LL (∼49) (Fig. 2*D*), showcasing the predicted structural diversity achieved through the simple chiral replacement of the acetamidated L amino acid at the *i* + 4 site with the D-amino acid version. We also performed a two-sample KS test on the 3D PSA data (Fig. S10), which further validated these *in silico* modeling results.

### Cell penetrability and uptake mechanisms of SRC2 peptide analogues

Given that the parameters calculated above are closely related to the subject molecules’ membrane permeability (33, 34, 35, 36), we were curious about the cell penetrability of our FTDR-stapled peptides and wondered if their increased flexibility, facilitated by the X_L/D_ amino acid and benzene-thiol linker, could allow for the adoption of more favorable conformations in hydrophobic microenvironments. Specifically, such dynamic structural adaptations may temporarily shield polar groups, aiding penetration through the hydrophobic core of cell membranes (37, 38, 39). The higher number of intramolecular hydrogen bonds in SRC2-LD or LL (23/20 for nHBD/nHBA *versus* 21/16 of RCM) (Table 2) may facilitate better mediation of these different conformations in highly flexible molecules consisting of multiple H-bonding sites. This idea is supported by the trend of decreasing PSA in hydrophobic environments (PSA = 690.1 for LD, 697.1 for LL, and 618.3 for RCM in water; PSA = 604.8 for LD, 646.1 for LL, and 570.5 for RCM in chloroform) (Fig. S9) (40, 41).

**Figure 3:**
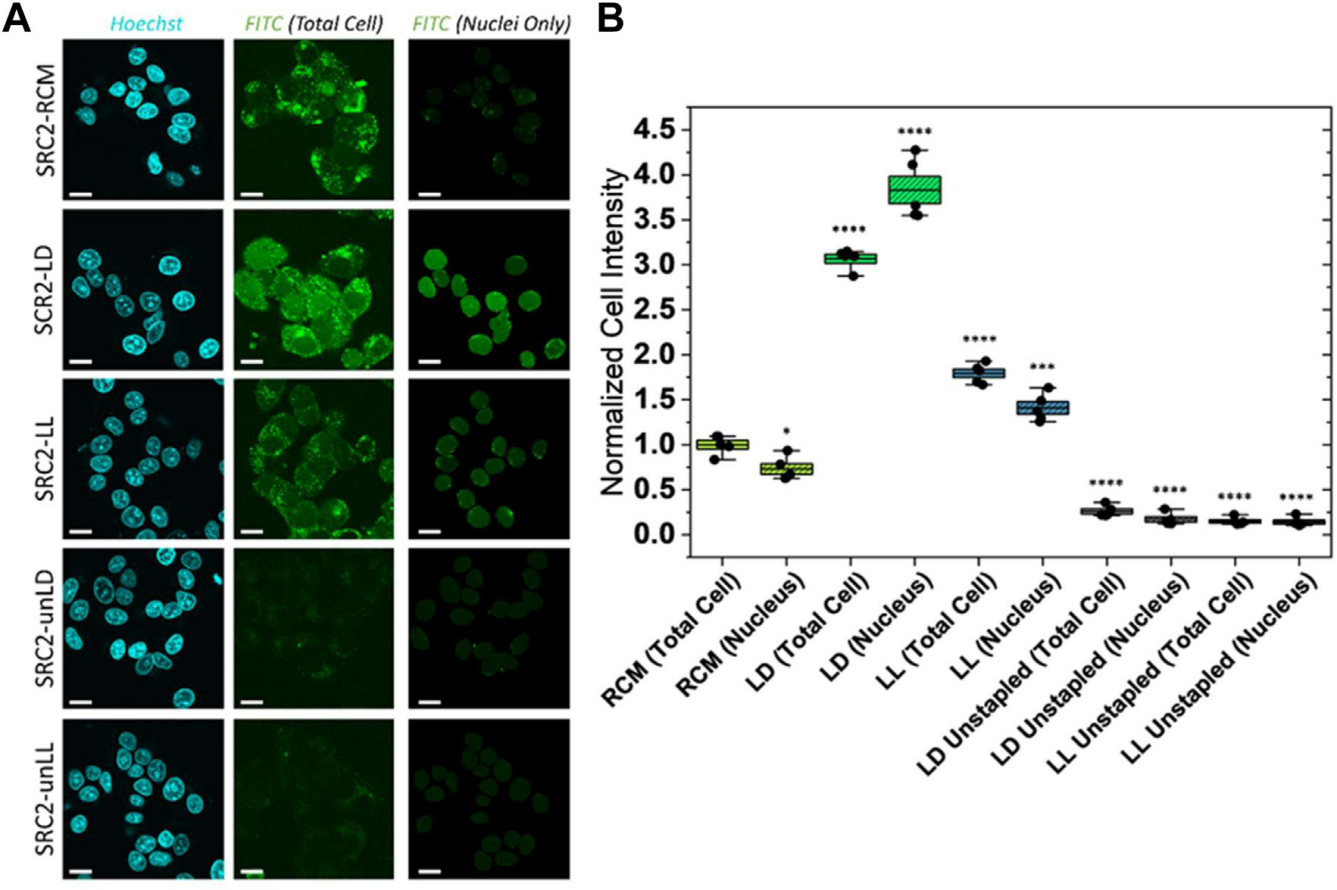
**Cellular uptake of stapled peptides.***A*, representative confocal images of live MCF-7 cells displaying 24 h cellular uptake of FITC-conjugated peptides. The *blue* channel represents Hoechst nuclear staining, while the *green* channels show FITC-labeled peptides for total cell (*middle*) and nuclear uptake (*right*), respectively. The scale bars represent 5 μm. *B*, quantification of peptide uptake with the plot showing the mean fluorescence intensity in the total cell and the nucleus, relative to SRC2-RCM (Total Cell) which was normalized to 1 as the control. Data represent n = 5 biological replicates. *Box* plots display the median of all samples, with overlaid individual points each representing an independent trial. Error bars indicate 95% confidence intervals, and boxes represent standard errors. An unpaired two-sided Welch’s *t* test was used to determine the statistical significance against SRC2-RCM (Total Cell), where ‘∗’ indicates a *p*-value of < 0.05, ‘∗∗∗’ indicates a *p*-value of < 0.001, and ‘∗∗∗∗’ indicates a *p*-value of < 0.0001. RCM, ring-closing metathesis; SRC2, steroid receptor coactivator 2.

We then set out to experimentally investigate the cellular uptake of FITC-labeled SRC2 peptides in a representative ER + cell line, MCF-7, using confocal microscopy. After a 24-h incubation, the FTDR-stapled peptides exhibited markedly better cell penetration than SRC2-RCM across the cytoplasm and the nucleus (Fig. 3). With the mean fluorescence intensity in SRC2-RCM treated total cells normalized to 1.0, the total cell uptake for SRC2-LL and SRC2-LD increased by approximately 1.79-fold and 3.07-fold, respectively (Fig. 3*B*). Similarly, the normalized nuclear uptake for SRC2-RCM, SRC2-LL, and SRC2-LD reached 0.73, 1.41, and 3.83, respectively. While the nuclear penetration appeared weaker for the RCM and LL stapled compared to their plasma membrane uptake, SRC2-LD is the only group which displayed an enhanced degree of nuclear penetrance, surpassing its already high level of cytoplasmic uptake.

To confirm that the FTDR stapling was the cause for the cell penetration of SRC2-LL/LD, we tested unstapled versions of these peptides (Fig. 3*A*, Table S2). Compared to the SRC2-RCM control, the LL unstapled and the LD unstapled displayed only 0.15-fold and 0.26-fold whole cell uptake, respectively, which are negligible (Fig. 3*B*). Such a substantial increase of membrane permeability endowed by the FTDR staple was unanticipated, challenging previously held beliefs that helicity, hydrophobicity, and rigidity were the major driving forces of the cellular uptake of peptides (4, 31, 42). Nevertheless, the significant boost in membrane penetration of FTDR staples over the hydrocarbon cross-link by RCM supports the theory that helicity alone is insufficient for cell penetration despite the common observation that more helical peptides are often more permeable than those of lesser structured conformations (4, 5, 6).

To investigate further into the mechanism of these peptides’ cellular membrane penetration, we blocked individual endocytosis pathways to observe which pathways may be used for peptide uptake. Previously, we have shown that other FTDR-stapled peptides use multiple different endocytosis pathways for cell uptake including caveolin-mediated (inhibited by nystatin), clathrin-dependent (suppressed by chlorpromazine), actin-polymerization mediated (blocked by cytochalasin D), and sulfated proteoglycan-related (downregulated by NaClO_3_) (25). In addition to these four pathways, we included two inhibitors of ATP-dependent transport (NaN_3_ and 2-deoxy-D-glucose) and two inhibitors of phagocytosis/macropinocytosis (wortmannin and 5-(*N*-Ethyl-*N*-isopropyl)amiloride (EIPA)) (43). We additionally tested uptake at 4 °C which is expected to slow all general forms of membrane trafficking.

**Figure 4:**
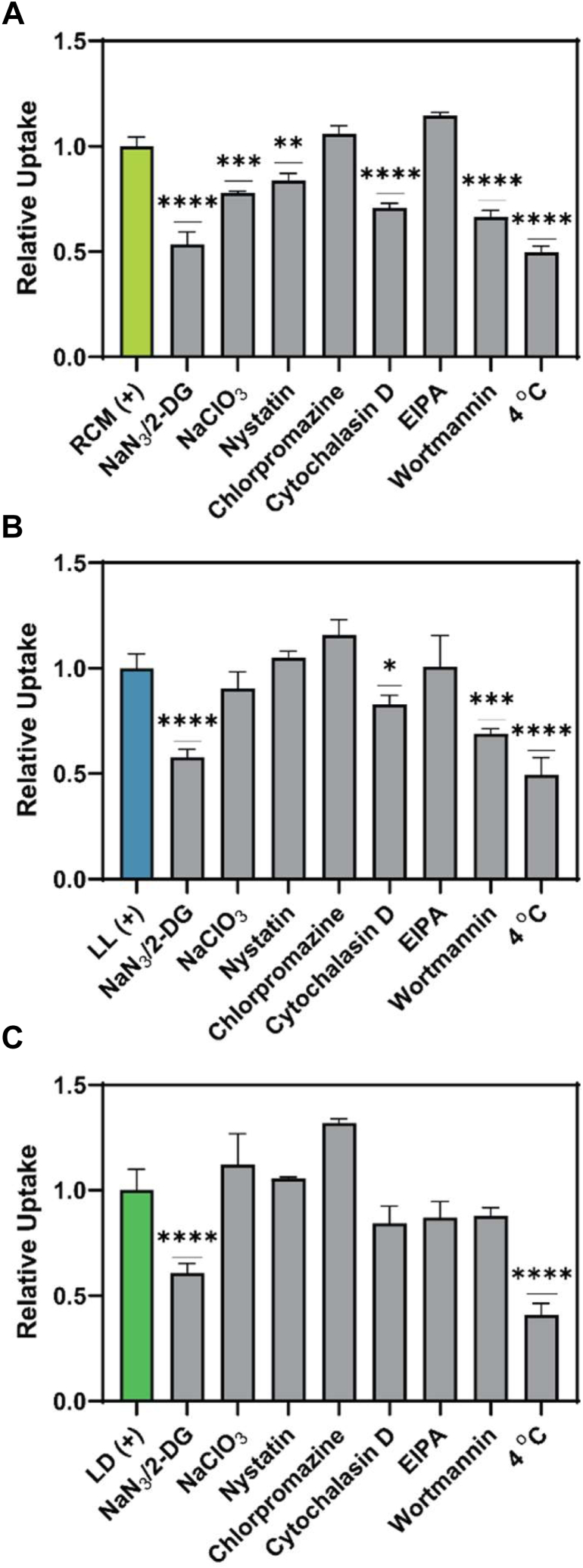
**Cellular uptake endocytic pathway investigation.** Plots represent relative uptake of (*A*) SRC2-RCM peptide, (*B*) SRC2-LL peptide, and (*C*) SRC2-LD peptide. MCF-7 cells were treated with small-molecule endocytic pathway blockers for a total of 5 h (1 h pretreatment and 4 h with 15 μM FITC-peptide). The final concentration of blockers used is as follows: NaN_3_ (sodium azide, 10 mM), 2-DG (2-deoxy-D-glucose, 30 mM), NaClO_3_ (sodium chlorate, 80 mM), nystatin (50 μM), chlorpromazine (5 μg/ml), cytochalasin D (10 μg/ml), EIPA (5-(N-ethyl-N-isopropyl)amiloride, 50 μM), wortmannin (200 nM). Data represent triplicate median fluorescent intensity (MFI) values normalized to uninhibited control. Error bars represent mean ± SD. An ordinary one-way ANOVA statistical analysis was used to determine significance to the uninhibited peptide group where ‘∗’ indicates a *p*-value of < 0.05, ‘∗∗’ indicates a *p*-value of < 0.005, ‘∗∗∗’ indicates a *p*-value of < 0.0005, and ‘∗∗∗∗’ indicates a *p*-value of < 0.0001. EIPA, 5-(*N*-Ethyl-*N*-isopropyl)amiloride.

All stapled peptides in this study showed the most impaired cell uptake upon disruption of general active transport. The uptake of SRC2-RCM was most affected by multiple inhibitors, with the most significant inhibition caused by NaN_3_/2-deoxy-D-glucose (2-DG) and 4 °C incubation leading to ∼ 50% inhibition, suggesting a high dependence on energy-dependent transport. Treatment with wortmannin and cytochalasin D also inhibited SRC2-RCM’s uptake, indicating a reliance on actin-mediated processes, specifically macropinocytosis/phagocytosis, consistent with reported findings (4, 6, 43). Slight decreases in uptake were observed with sodium chlorate and nystatin treatment, indicating a partial reliance on sulfated membrane-bound proteoglycans and caveolin-mediated endocytosis, respectively (4, 6, 43). SRC2-LL showed inhibited penetration with NaN_3_/2-DG and at lower temperatures, indicating a high reliance on active transport processes. Additionally, partial inhibition was also observed by cytochalasin D and wortmannin, implicating the involvement of actin-mediated and macropinocytosis pathways, respectively (Fig. 4*B*). Macropinocytosis has been recently established as a key pathway of cellular uptake for peptidomimetics and cyclic peptides of similar size (43), which SRC2-RCM and SRC2-LL peptides appear to partially use. SRC2-LD‘s uptake was primarily affected by energy-dependent uptake (NaN_3_/2-DG, and 4 °C, Fig. 4*C*) but was generally uninhibited by the disruption of the other internalization pathways. A slight decrease was observed with inhibitors related to actin-polymerization (cytochalasin D) and macropinocytosis (wortmannin and EIPA), which may collectively contribute to SRC2-LD peptide’s active membrane transport. Chlorpromazine treatment led to an increase in uptake for all three stapled peptides, a phenomenon observed in other reported cell-penetrating peptides (4, 44, 45). Additionally, EIPA treatment resulted in either increased peptide uptake or an insignificant decrease for SRC2-LD (4, 6). Representative histograms for the flow cytometry data can be found in the supplementary (Figs. S11–S13).

Interestingly, SRC2-RCM was observed to use the widest range of endocytic pathways yet showed significantly reduced uptake compared to SRC2-LD and SRC2-LL. This suggests that although fewer transport mechanisms are used by the FTDR peptides, their efficiency in traversing these pathways could be higher. The observation that the most potent inhibitor conditions, ATP-depletion (NaN_3_/2-deoxy-D-glucose) and 4 °C incubation only resulted in an approximate 50% decrease of cell permeability for the stapled peptides suggest that passive membrane transport is likely involved, as passive diffusion is less sensitive to lower temperatures than active transport processes (46, 47, 48). We cannot rule out the possibility that some of these SRC2 peptides, if not all, can passively transport across the membrane, similarly to the natural macrocyclic peptide, Cyclosporin A, which has been shown to passively diffuse through the plasma membrane due to its flexibility allowing high conformational diversity and adaptability to the microenvironment (49, 50, 51).

To further evaluate the potential involvement of passive transport in the uptake of FTDR peptides, we performed a cellular uptake competition assay. MCF-7 cells were incubated with 15 μM FITC-labeled peptides in the presence of 100-fold excess of unlabeled peptide (Fig. S14). The results showed that the uptake of SRC2-LD and SRC2-LL peptides was not significantly affected by the presence of excess unlabeled peptide, suggesting a nonsaturable uptake mechanism under these conditions. This finding indicates that the intracellular transportation of SRC2 peptide may not be molecule-specific, which could be consistent with some general uptake processes such as fluid-phase endocytosis or passive diffusions. Collectively, these observations highlight the complexity of cellular permeability for these FTDR peptides, implicating the potential involvement of both energy-dependent and energy-independent transport mechanisms. Representative histograms have also been included in the supporting information, presented as Fig. S15.

Parallel to these assays, all endocytic inhibitors were separately tested for cytotoxicity on MCF-7 cells and were revealed to have little effect on cell viability (Fig. S16). We also performed an ATP depletion assay of the NaN_3_/2-DG inhibitor-treatment on MCF-7 cells to validate that ATP levels were being significantly impacted over the 5 h total treatment which confirmed a ∼98% decrease in ATP levels compared to vehicle-treated cells (Fig. S17).

### Phenotypic evaluation in ER + BC cells

**Figure 5:**
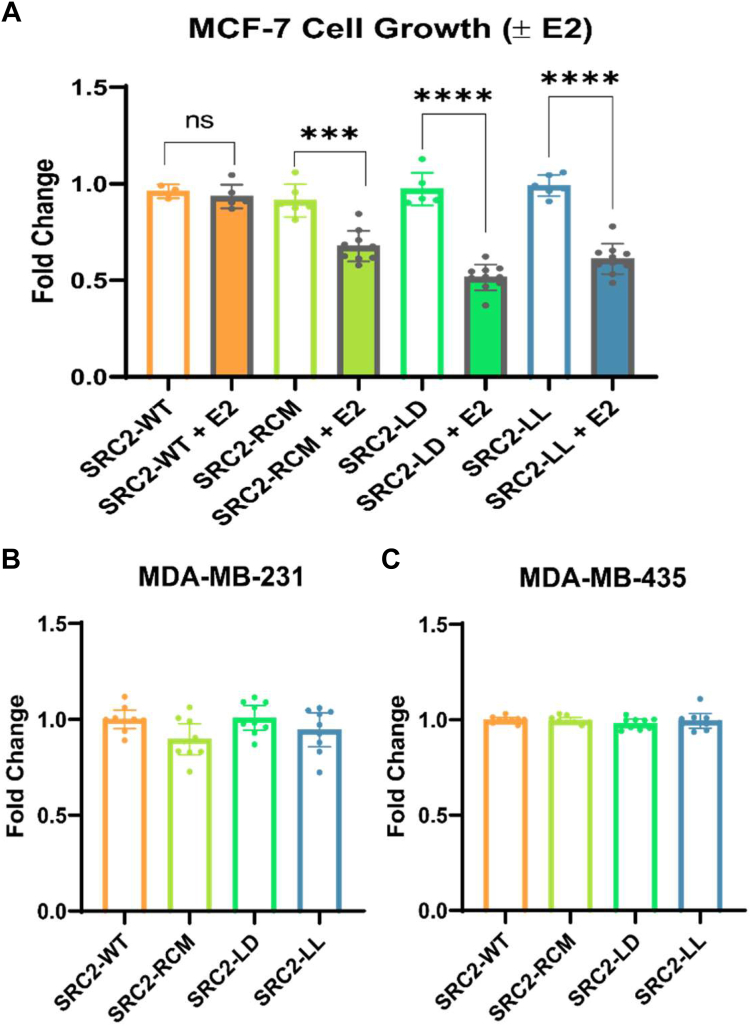
**Effects of stapled SRC2-peptides on cell proliferation.***A*, cell viability of MCF-7 cells in the presence or absence of 10 nM E2. Cells were treated with 15 μM peptide. Data represent cell counts on the fifth day post treatment, shown as mean ± SD, and normalized as fold change relative to vehicle ± E2. For E2-supplemented groups, data represent biological duplicates (n = 6, 10) while for groups without E2, data represent a single experiment (n = 3, 6). *B* and *C*, effects on cell proliferation in negative control cell lines treated with 15 μM peptide. Data represent biological triplicates (n = 9), measured using cell counts on the fifth day post treatment, shown as mean ± SD, normalized to vehicle treatment. *B*, MDA-MB-231 triple-negative breast cancer cells. *C*, MDA-MB-435 ER-negative cells. Statistical significance was determined using an unpaired two-sided Welch’s *t* test. ‘∗∗∗’ indicates a *p*-value of < 0.005 and ‘∗∗∗∗’ indicates a *p*-value of < 0.0001. ER, estrogen receptor; SRC2, steroid receptor coactivator 2.

To evaluate the phenotypic efficacy of our highly permeable peptides, we treated ER + MCF-7 BC cells with each peptide upon E2 addition. Since these peptides were modeled on a nonlethal coactivator interaction, we expected an effective candidate would counteract the proliferative effect of E2 without resulting in any significant cytotoxicity on the cell population. The antiproliferative effect of our stapled peptides was thereby assessed after a 5-day incubation with E2-treated MCF-7 cells. Using an incubation concentration of 15 μM, SRC2-LD and SRC2-LL treatment led to a significant decrease of 48% and 38% of the total cell population, respectively, with SRC-RCM treatment resulting in a 33% reduction compared to vehicle (Fig. 5*A*). In the absence of estradiol stimulation, none of the peptides led to any significant decrease of MCF-7 cell proliferation compared to the control (Fig. 5*A*). To ensure that these observed effects were from the disruption of the ERα interaction, we repeated the proliferation assay using two ER-negative cell lines, MDA-MB-231 and MDA-MB-435 (52, 53), which displayed minimal growth inhibition upon treatment with all peptide groups (Fig. 5, *B* and *C*).

**Figure 6:**
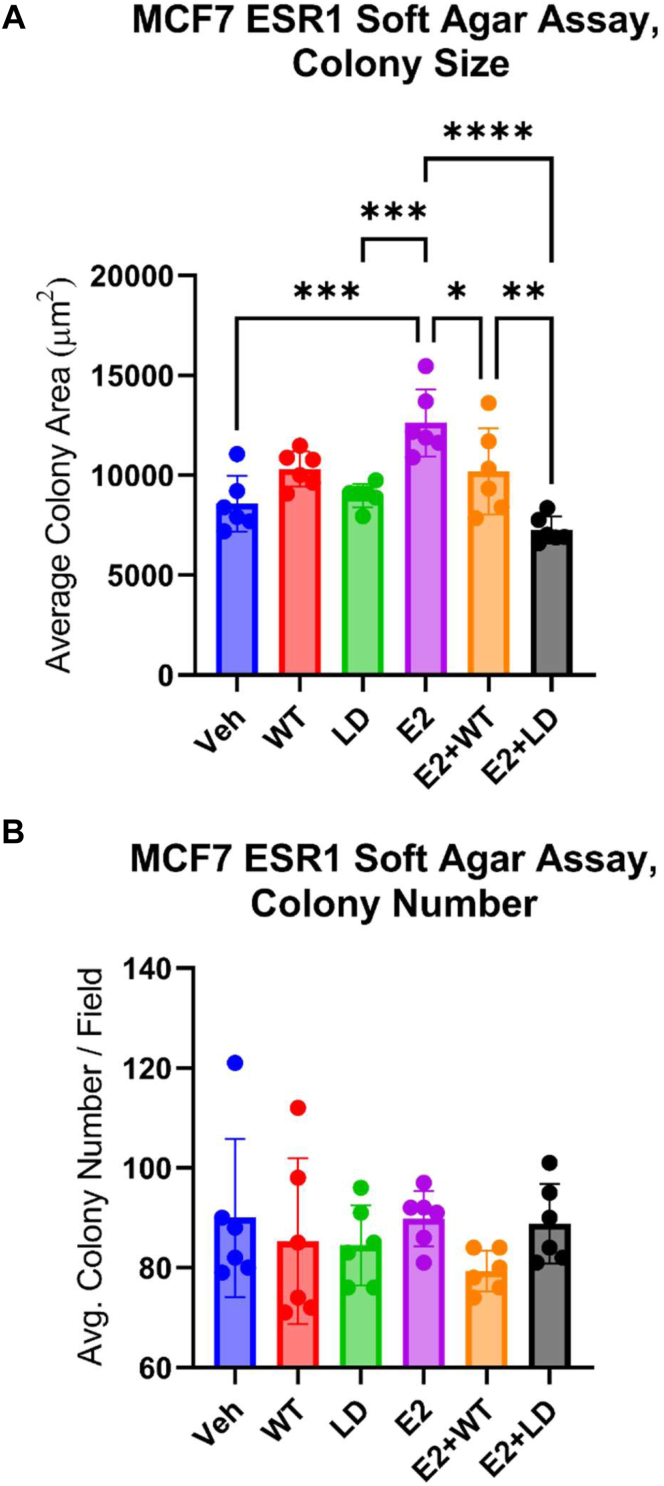
**Soft agar colony formation assay.** (*A*) soft agar average colony size and (*B*) average colony formation in MCF-7 ER + cells assessed in the presence of vehicle (EtOH), E2 (1 nM), and the indicated peptide treatments (WT, LL, or LD) at 20 μM. Graphed data represent the mean ± SD (n = 6). An ordinary one-way ANOVA was used to assess statistical significance where ‘∗’ indicates *p* < 0.05, ‘∗∗’ indicates *p* < 0.01, ‘∗∗∗’ indicates *p* < 0.001. ER, estrogen receptor.

**Figure 7:**
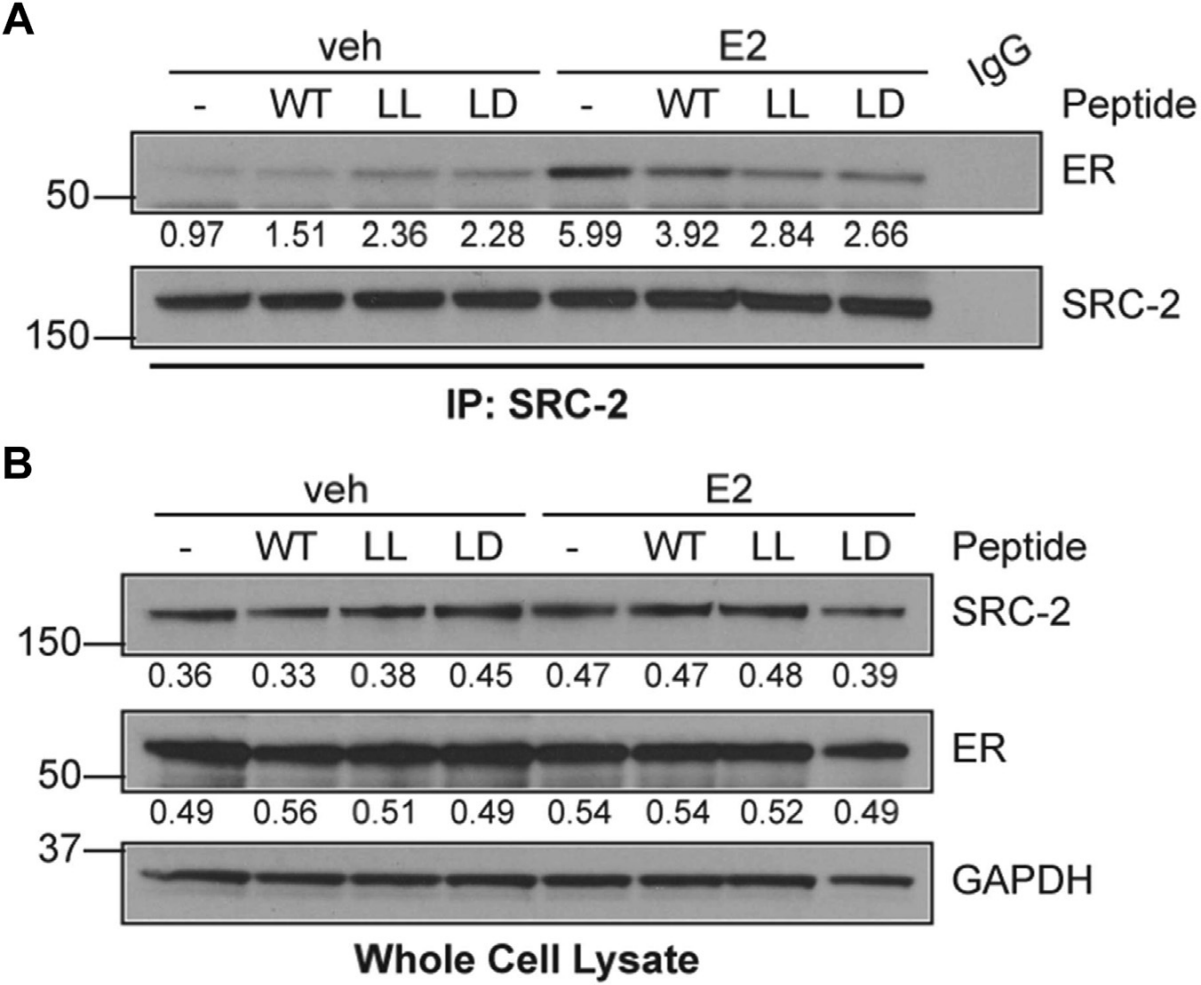
**Effect of SRC2 peptides on the cellular estrogen receptor (ER) interaction with coactivator SRC-2.***A*, Co-immunoprecipitation of ER by SRC-2 in MCF-7 cells. Cells were pretreated with 20 μM of WT, LL, or LD peptide, followed by E2 (1 nM) treatment for 30 min. Quantification shown as ER divided by SRC-2. All signals have been background corrected. *B*, whole cell lysate. Quantification shown as ER or SRC-2 divided by GAPDH (loading control). All signals have been background corrected. SRC2, steroid receptor coactivator 2.

This ER-selective inhibitory effect on cell growth was also observed in soft agar assays. While colonies between treated and untreated groups remained consistent in overall cell number, a notable difference in colony size was evident. Specifically, the average colony sizes for the vehicle-treated group, SRC2-WT, and SRC2-LD were 12,620 μm^2^, 10,193 μm^2^, and 7254 μm^2^, respectively (Fig. 6). This decrease in colony sizes in peptide-treated groups represents a 42% downregulation for SRC2-LD compared to the vehicle and a 19% downregulation for SRC2-WT compared to the vehicle. We found that SRC2-LD was the most effective disruptor of proliferation, likely owing to its high plasma and nuclear membrane penetrability. Treatment with SRC2-LD, but not SRC2-LL was also shown to interfere with ER-pull-down by SRC2 antibody in a co-immunoprecipitation (Co-IP) assay, along with a minor reduction of SRC2 in the MCF-7 whole cell lysate (Fig. 7). Quantification of these Co-IP assays from multiple independent experiments can be found in the supporting information (Fig. S18). As expected, no ER degradation was observed for either FTDR peptide, indicating that the mechanism involves ER modulation, consistent with other reported constructs (18). This observation further confirmed the SRC2-LD peptide’s cellular growth inhibition effect was due to its efficient disruption of the cellular estrogen receptor-coactivator (ER-SRC2) interaction.

### Affinity to the ligand-binding domain of ERα

Lastly, we performed fluorescence anisotropy (FA) to assess the binding capacity of the SRC2 peptides to ERα-LBD. Unexpectedly, our FTDR-stapled peptides displayed comparable affinity to SRC2-WT but slightly weaker binding than SRC2-RCM, with a 1.2-fold and 1.5-fold increase in *K*d for SRC2-LD and SRC2-LL, respectively (Fig. S19). Despite this, SRC2-LL demonstrated comparable antiproliferative effects and SRC2-LD displayed even more enhanced antiproliferation than SRC2-RCM, given the growth inhibition assay results (Fig. 5*A*) highlighting the importance of cell penetrability being able to compensate for its weaker receptor binding. The poor cell penetration, particularly the weaker nuclear uptake of SRC2-RCM, may limit peptide accumulation inside the cellular compartments necessary for interaction with the ER. This suggests that the superior uptake of SRC2-LD compensates for its weaker binding affinity, leading to a more significant decrease in proliferation. Rapid entry into the cell and the nucleus appears to be crucial for phenotypic effect in this ER and coactivator interaction, emphasizing the importance of membrane penetrability.

## Discussion

The interaction between the nuclear receptor ERα and the coactivator SRC2 represents one of a typical disease-related protein-protein interaction, which regulates the onset and relapse of ER + breast cancer that remains lethal and drug-resistant. The large interface between ERα and SRC2 along with its allosteric and plastic nature makes it ideal to be targeted by peptides rather than small molecules. Nevertheless, most peptide-based agents were limited to *in vitro* applications due to their mediocre membrane permeability. Even with the recent rise of RCM-mediated hydrocarbon stapling, peptide derivatives with significant rigidity and improved α-helical folding have been reported to still suffer from limited cellular uptake. In most instances, the incorporation of additional cell penetrating peptides or positively charged amino acids is necessary which nonetheless may compromise cell membrane integrity.

The previous success of our FTDR-stapled peptides in achieving general cell uptake prompted us to design and develop a class of FTDR-stapled SRC2 peptide derivatives. Typically, FTDR-stapled LL and LD peptide probes targeting other PPIs exhibit comparable properties and biological activities, including cellular uptake. Surprisingly, the SRC2-LD peptide proved to be a more effective probe than all the L-amino acid containing, SRC2-LL. First of all, SRC2-LD exhibited chameleonic properties in solvents of varied hydrophobicity due to its high flexibility. This was demonstrated by largely varied α helicity in the related experiments and was further validated by the lower LogP but higher PSA, R_gyr_, nHBD/nHBA, and nIMHB values calculated *in silico*. Through conformational sampling, we illustrated the diverse set of predicted conformations that SRC2-LD can adopt depending on the local microenvironment. Particularly, the replacement of L-to D-chirality at the *i* + 4 site appeared to promote α-helical folding in more hydrophobic environments, to a much larger extent than the original SRC2-LL counterpart. Compared to SRC2-LL and the traditionally hydrocarbon stapled SRC2-RCM, SRC2-LD displayed much more significant uptake into the cytoplasm and nucleus of the ER + BC cell line, MCF-7. This observation suggests the D isomerization at the *i* + 4 site for FTDR-stapling could yield peptides with high inherent permeability without the need for additional cell-penetrating or nuclear localization tags.

We have demonstrated that FTDR-stapled peptides use distinct pathways for cellular uptake for X_L_/X_D_ containing peptides. Although the SRC2-LL peptide acted similarly to SRC2-RCM, primarily using ATP-dependent processes and actin-mediated/macropinocytic processes for membrane penetration. SRC2-LD appeared to adopt certain unknown pathways that are also ATP-dependent such as the possible utilization of membrane receptor-mediated active transportation (54, 55). All of these stapled peptides likely partake in passive diffusion as well. Despite the increased flexibility which led to reduced helicity and slightly weaker target binding, FTDR peptides displayed improved antiproliferative effects compared to the WT and the RCM stapled ones. This signifies that provided there is sufficient target affinity, high cellular uptake (and nuclear penetration for this ERα – SRC2 interaction) can compensate for weaker binding leading to more impactful phenotypic activity.

**Figure 8:**
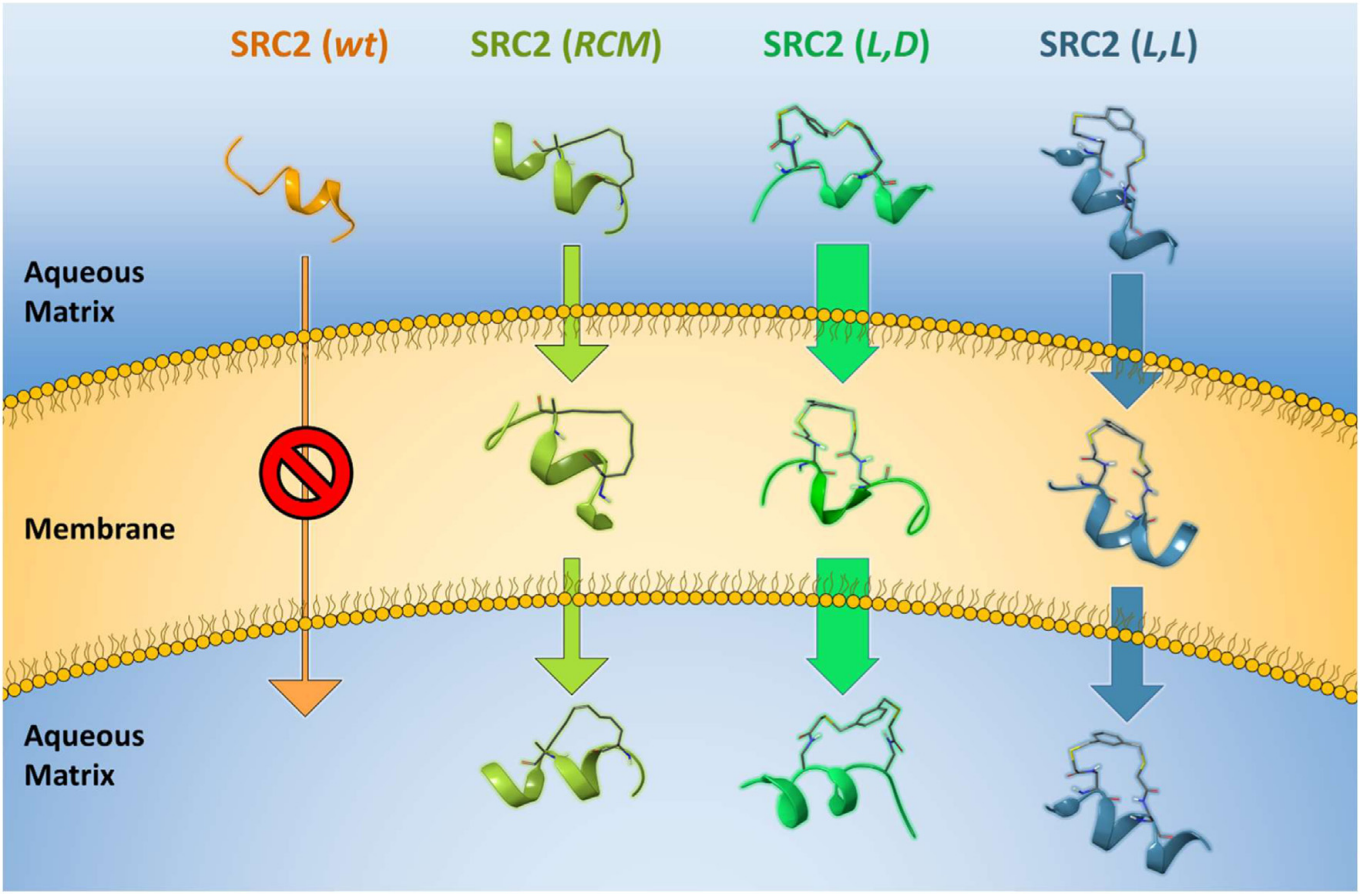
**Representative illustration of plasma membrane trafficking of SRC2 peptide analogues.** Peptide images represent prevalent structures modeled in water (aqueous matrix) and chloroform (membrane), illustrating the differences in flexibility between the groups and relative cellular uptake. SRC2-LD displays the highest chameleonicity, effectively folding into a more compact conformation in the hydrophobic membrane, effectively shielding its polarity. SRC2, steroid receptor coactivator 2.

In conclusion, this work illustrates a special case where peptides stapled using different chemical methods to target the estrogen receptor-coactivator interaction exhibit strikingly distinct membrane permeability (Fig. 8). Contradictory to the central dogma that conformational structural factors such as α-helicity, rigidity, and lipophilicity are crucial for cellular uptake, our FTDR-stapled peptides challenge these notions by showing that their innate flexibility led to significantly enhanced permeability to not only the cytoplasmic membrane but also the nuclear envelope (Fig. 8). Our findings suggest there is still an incomplete understanding of the complicated factors that control the membrane permeability of macromolecules such as peptides. A major part of the observed activity enhancement was due to the L to D isomerization at the *i* + 4 site of the FTDR staple. Although D-amino acid inclusion has been documented to modulate the structure and functions of peptides (*e.g.*, improved biostability and reduced immunogenicity) (56, 57, 58, 59, 60), it usually yields negative effects on α-helical folding and other associated properties (61). This work serves as a benchmark for the potential application of D-amino acids in the rational design of FTDR-stapled peptides to promote α-helical conformations and enhancing membrane permeability. To the best of our knowledge, this represents the first demonstration of such an effect by D-amino acids.

The primary weakness of the FTDR-stapled peptides presented here lies in their slightly weaker binding affinity compared to the RCM-stapled control, likely due to their high structural flexibility. This flexibility detracts from the constrained and rigid helical structure hindering the peptides’ ability to maintain a constructive binding interaction with their target. Regarding the limitations of our methods, although FITC-labeling is a useful and robust tool for assessing cellular uptake, it sometimes may not identically represent the behavior of unlabeled peptides. Our future work will focus on optimizing the binding affinity of these FTDR-stapled SRC2 peptides through methods such as affinity maturation in order to achieve improved efficacy.

## Experimental procedures

### Peptide synthesis

Peptides were synthesized on rink amide resin (Aapptec) using standard Fmoc solid-phase procedures (62), incorporating either S_5_ or X_L/D_ for respective, RCM and FTDR stapling performed as previously reported (25, 28). Peptides were synthesized using a CS136X automatic peptide synthesizer (CSBio) which uses standard Fmoc-based synthesis using the following: 4 eq. of each amino acid, 4 eq. of 2-(1H-benzotriazole-1-yl)-1,1,3,3-tetramethyluronium hexafluorophosphate, and 8 eq. of diisopropylethylamine (DIPEA) all solubilized in dimethylformamide (DMF). Each addition of amino acid to the peptide chain was performed at room temperature for 45 min of shaking followed by several rounds of DMF washing. N-terminal Fmoc deprotection was performed twice using 20% piperidine in DMF before subsequent amino acid conjugation. FITC was conjugated to the N-terminal β-alanine using 7 eq. of FITC and 14 eq. of DIPEA in DMF. After fluorophore conjugation the beaded peptide was handled in the dark to avoid photobleaching. Peptides that were not FITC-conjugated were capped using excess acetic anhydride and DIPEA. The resin was washed with DMF and methylene chloride several times before drying under vacuum.

Peptide side chain deprotection and bead cleavage were accomplished through incubation with cleavage cocktail–TFA/triisopropylsilane/H_2_O-95/2.5/2.5 (v/v/v). Cleavage occurred with gentle shaking at room temperature for 2 h. After cleavage, TFA was evaporated using nitrogen gas before the addition of cold diethyl ether to precipitate the peptide product. Peptides were then centrifuged, and the ether supernatant was decanted. The peptide precipitate was washed an additional two times with cold diethyl ether before being dried under vacuum. Selected peptides were then FTDR stapled without purification while both RCM stapled and WT peptides were dissolved in minimal volumes of methanol/water then purified by HPLC.

### General procedures for peptide stapling

On-bead olefin metathesis was performed as previously reported by Kim *et al.* (28) Briefly, the resin-bound peptide was treated with freshly made Grubbs’ first-generation catalyst (0.5 eq. to resin) in DMF. The resulting resin mixture was gently shaken using a rotisserie shaker at room temperature for 2 h. The reaction mixture was drained from the vessel, and the reaction was repeated an additional two times using fresh catalyst. The resin was washed multiple times using DMF and dichloromethane and dried under vacuum. The follow-up N-terminal FITC addition and peptide cleavage were performed as described above.

FTDR stapling was performed as done previously (25). Briefly, 1,3-benzenedimethanethiol (6 eq.), NaOH (12 eq.), and DMF were mixed at room temperature for 5 min to activate the thiol linker, before mixing with water solubilized (L,L) or (L,D) peptides (1 eq.). Peptide reaction mixtures were incubated at 37 °C overnight (∼16 h). The following morning, a small aliquot was removed from each reaction and quenched with acetic acid (pH < 5) before running on LC-MS (Agilent 1260 Infinity II) to confirm completion of stapling. After confirmation, stapled product was precipitated from the solution with cold diethyl ether and dried before subsequent HPLC purification.

### HPLC purification

All peptides were purified with a 1525 HPLC (Waters) equipped with an XBridge Prep C18 OBD column. The solvent system used was mobile phase A (0.1% TFA in water) and mobile phase B (0.1% TFA in acetonitrile) at a flow rate of 10 ml/min. Absorbances were read at 210 nm and 254 nm. Peptides were solubilized in water and injected in 5 ml injection volumes. The gradient method is as follows: initially, 90% A and 10% B for 5 min to load peptide onto the column. Then, an increase to 25% B over an additional 5 min. Finally, an increase from 25% to 55% B over a 20 min gradient during which time the product is eluted, corresponding to the major peak.

### Circular dichroism

CD spectra were obtained using a J-815 CD spectrometer (Jasco) using the wavelength range of 190 to 260 nm. Spectra were collected in a 10 mm quartz cuvette and each spectrum represents the accumulation of three solvent-blank subtracted runs. Data were collected using the following system parameters: bandwidth of 1.00 nm, digital integration time of 2 s, pitch of 1.0 nm, and a scan speed of 50 nm/min. Data underwent minimal adaptive smoothing, and all the related spectra were taken at room temperature under inert nitrogen flow. Peptides were solubilized to 50 μM in either Milli-Q water, 45 mM sodium phosphate buffer (pH 7.4), 30 mM SDS (pH 7.4), or 50% (v/v) TFE. Percentage of α helicity was calculated based on the ellipticity value at 222 nm as done previously (25, 63), following the equation α helicity $\left(\%\right)=\left({\left[ϴ\right]}_{\text{obs}222}-{\left[ϴ\right]}_{0}\right)/\left({\left[\;ϴ\right]}_{\max\;222}-{\left[ϴ\right]}_{0}\right)$ where [ϴ]_max222_ = (−44,000 + 250T) (1-x/n), [ϴ]_0_ = −2220 – 53T, T = 20 °C, x = 3, n = number of residues (25, 63).

### Cell culture and general reagents

STR authentication for cell lines was performed by American Type Culture Collection (August 2021). All cell lines were tested for *mycoplasma* (e-Myco, Bulldog Bio) prior to initiation of experiments. MCF7 cells were cultured in Dulbecco's modified Eagle's medium (DMEM) (Corning, #10013CV) containing 5% fetal bovine serum and 1% penicillin-streptomycin. For experiments with hormone treatment (*i.e.*, E2), cells were hormone-starved in phenol-free modified improved minimum essential medium (Gibco, # A1048801) containing 5% dextran-coated charcoal for 16 h prior to treatment. Estradiol (E2; Sigma-Aldrich) stocks were prepared in ethanol (EtOH). SRC-2 WT, SRC-2 LL, and SRC-2 LD stapled peptide stocks were prepared in PBS buffer.

### Confocal imaging

All confocal imaging, including the relating fluorescence intensity analysis of individual cells was performed as previously reported (25, 64). An Olympus FV3000 confocal laser scanning microscope was used to gather all the related cellular images. Excitation at 405 nm and 488 nm were adopted for detection of Hoechst dye and FITC, respectively, and was achieved through laser excitation (Coherent OBIS). Cells were imaged in a physiological mimicking imaging buffer as described previously (25, 64). Image acquisition was conducted across five independent biological replicates (n = 5). Images were processed and analyzed using ImageJ (NIH). Cellular uptake of FITC-labeled peptides was quantified by measuring the mean fluorescence intensity within defined cellular regions (total cell or nucleus).

### Cell penetration assay

MCF-7 cells were seeded on 35 mm glass optical dishes at a density of 30,000 cells and were incubated overnight at 37 °C and 5% CO_2_. FITC-labeled SRC2 peptide analogues were added and incubated for 24 h at a final concentration of 15 μM. Hoechst 33342 (1 μg/ml in PBS) was used for nuclear staining and propidium iodide was used as a cell viability stain.

### Cell penetration pathway study

MCF-7 cells were seeded in 24-well plates in the culture medium and incubated overnight at 37 °C and 5% CO_2_. The following day, media were replaced with deprivation medium (DMEM phenol-red free, serum-free, and insulin-free) supplemented with small-molecule endocytic inhibitors for 1 h: NaN_3_ (10 mM), 2-DG (30 mM), NaClO_3_ (80 mM), nystatin (50 μM), chlorpromazine (5 μg/ml), cytochalasin D (10 μg/ml), EIPA (50 μM), and wortmannin (200 nM). A separate plate was preincubated in a 4 °C fridge for 1 h with prechilled media for use as cold temperature treatment. FITC-labeled peptide was then added to a final concentration of 15 μM and incubated for an additional 4 h. The media were removed, and wells were washed gently with PBS. Cells were dissociated from the wells by Trypsin (0.25%) digestion for 5 min before being quenched with cold serum-free medium. Cells were transferred to 2 ml Eppendorf tubes and pelleted by centrifugation at 300*g* for 3 min at 4 °C. The supernatant was decanted, and cells were resolubilized in cold PBS and stored on ice. Immediately before running on a BD Accuri C6 flow cytometer, 0.4% Trypan blue was added to 10% (v/v). Approximately 10,000 events were collected for each cell sample on the flow cytometer. FlowJo v10.8.1 Software (https://www.flowjo.com) (BD Life Sciences) was used for data analysis. The median fluorescence intensity of gated live-cells (n ∼ 10,000) was used to quantitatively determine FITC-peptide that was uptaken into cells. Data represent triplicate wells for each condition.

### Cell proliferation assay

MCF-7, MDA-MB-231, or MDA-MB-435 cells were seeded in 96-well plates at ∼2000 cells per well in DMEM (10% fetal bovine serum with phenol red and 1 μg/ml human recombinant insulin). After overnight attachment, media were replaced with peptide (5, 10, and 15 μM) and E2 (10 nM) supplemented media and incubated at 37 °C and 5% CO_2_. After 5 days, Cell numbers were counted under the microscope and the proliferation level was determined relative to vehicle control.

### Co-IP assay

Cells were harvested in erythrocyte lysis buffer [50 mM Hepes, 0.1% NP-40, 250 mM NaCl, 5 mM EDTA, 1X complete protease inhibitors (Roche), 1X PhosSTOP (Roche), and supplemented with 1 mM PMSF, 1 mM NaF, 0.5 mM Na_3_PO_4_, 25 mM BGP, and 20 μg/ml aprotinin]. Subsequently, 1000 μg lysate (1 mg/ml) was incubated with 1 μg of the indicated antibody overnight at 4 °C. Immunocomplexes were isolated with protein G agarose (Roche) for 2 h at 4 °C. Resin was collected and washed three times with cold erythrocyte lysis buffer. Immunocomplexes were eluted with sample buffer, resolved by SDS-PAGE, and analyzed by Western blot.

### Immunoblotting

Antibodies used: SRC-2 (D2X4M, Cell Signaling), ERα (F-10, Santa Cruz Biotechnology), GAPDH (0411, Santa Cruz Biotechnology), goat anti-rabbit IgG-HRP (Bio-Rad), and goat anti-mouse IgG-HRP (Bio-Rad). Blots were developed with enhanced chemiluminescence using Super Signal West Pico Plus Chemiluminescence Substrate (Pierce) and imaged by film.

### Soft agar assay (anchorage independent growth)

Cells were seeded (1.5 × 10^4^ cells/well) in 1X sterile low melt agarose (Life Technologies) containing 5% dextran-coated charcoal and the appropriate treatment. Soft agar assays were allowed to proceed for 14 to 18 days at 37 °C. Afterward, cell colonies were stained with 0.005% crystal violet in PBS. Colony number and colony size were analyzed with ImageJ (Wayne Rasband, National Institutes of Health, http://rsbweb.nih.gov/ij/). Data are presented as the average ± SD of six independent measurements.

### Fluorescence anisotropy

Saturation FA assays were performed as described previously (65). All FA assays were performed on a Synergy Neo2 plate reader (Agilent Technologies) using 490 nm excitation and 520 nm emission filters. FA measurements were taken in black nonbinding surface Corning 3650 96-well plates, loaded with 40 μl of assay solution per well. Assays were performed using 10 mM Hepes buffer, pH 7.4, containing 50 mM EDTA, 150 mM NaCl, and 0.005% Tween-20. ERα ligand-binding domain in buffer was serially diluted to give final plate concentrations ranging from 0.4 to 7500 nM. Briefly, 17β-Estradiol was serially diluted to give final plate concentrations at four equivalents to ERα. The final plate concentration of each FITC-conjugated peptide was 50 nM in all wells. All aqueous solutions were prepared using deionized water collected from a Millipore water purification system. The experiments were performed in duplicate and replicated, and sigmoidal concentration-response curves were fitted to the data using GraphPad Prism 8 software (https://www.graphpad.com) (GraphPad Software, USA).

### Computational modeling

Maestro (Schrödinger Release 2022–13.4: Maestro, Schrödinger LLC, New York, NY) was used to perform all computational modeling. The initial peptide structure was obtained from the crystal structure of SRC2-RCM bound to the ligand-binding domain of Erα (Protein Data Bank: 5DXE) (27), wherein the dithiol benzene linker conjugated to the amide-containing side chains of our X_L/D_ residue took the place of the olefin staple present in the crystal structure. Conformational sampling was performed using the MacroModel Conformational Search program utilizing algorithms for both Mixed torsional/low-mode sampling (LMOD) and Monte-Carlo Multiple Minimum torsional sampling (MCMM) methods. The OPLS4 force field (default parameters) was equipped with both water and chloroform implicit solvent systems. Extended torsion sampling was performed with a maximum number of steps set to 10,000 and 150 steps per rotatable bond. The energy window for saving a structure was 21.0 kJ/mol and redundant structures were eliminated by a maximum atom deviation cutoff of 1.5 Å. All generated structures were checked for correct chirality. QikProp in Maestro was used to determine the following: LogP, 3D PSA, molecular volume, and the nIMHB) using default parameters. Structures were exported to PyMOL (The PyMOL Molecular Graphics System, Version 2.0 Schrödinger, LLC) to determine the R_gyr_. The total number of conformations generated and analyzed from MCMM and LMOD methods were as follows: 706 for RCM in water, 1404 for LD in water, and 1266 for LL in water; 608 for RCM in CHCl_3_, 567 for LD in CHCl_3_, and 393 for LL in CHCl_3_.

## Data availability

All data are contained within the manuscript and the supporting information. Any additional raw data is available upon request.

## Supporting information

This article contains supporting information.

## Conflict of interest

The authors declare that they have no conflicts of interest with the contents of this article.
