# Supplementary material for: Flexible fluorine-thiol displacement stapled peptides with enhanced membrane penetration for the estrogen receptor/coactivator interaction
Source: J Biol Chem. 2024 Nov 13;300(12):107991. doi: 10.1016/j.jbc.2024.107991 (PMC11667158; doi:10.1016/j.jbc.2024.107991)
Supplement: Supporting information [file mmc1.docx]

**Flexible Fluorine-Thiol Displacement Stapled Peptides with Enhanced Membrane Penetration for the Estrogen Receptor/Coactivator Interaction**

**Supporting Information**

Robert Maloney^1,§^, Samuel L. Junod^2,§^, Kyla M. Hagen^3, 4^, Todd Lewis^1^, Changfeng Cheng^5, 6^, Femil J. Shajan^1^, Mi Zhao^1^, Terry W. Moore^5^, Thu H. Truong^3, 4^, Weidong Yang^2,^*, Rongsheng E. Wang^1,^*

1. Department of Chemistry, Temple University, Philadelphia, Pennsylvania, USA
2. Department of Biology, Temple University, Philadelphia, Pennsylvania, USA
3. Department of Biochemistry, Molecular Biology and Biophysics, Masonic Cancer Center, University of Minnesota, Minneapolis, Minnesota, USA
4. Masonic Cancer Center, University of Minnesota, Minneapolis, Minnesota, USA
5. Department of Pharmaceutical Sciences, College of Pharmacy, University of Illinois at Chicago, Chicago, Illinois, USA
6. Current Address: Arrowhead Pharmaceuticals, Madison, Wisconsin, USA

^§^ These authors contributed equally.

* To whom correspondence should be addressed:

Email: [weidong.yang@temple.edu](about:blank), [rosswang@temple.edu](about:blank)

**Table S1. Summary of the prepared SRC2 peptides including LC-MS characterizations**

**Table S2. Cellular uptake comparison of stapled and unstapled peptides.** Fluorescence intensity data is normalized to SRC2-RCM (Total cell) mean ± SD value. N represents the total number of cells analyzed.

**
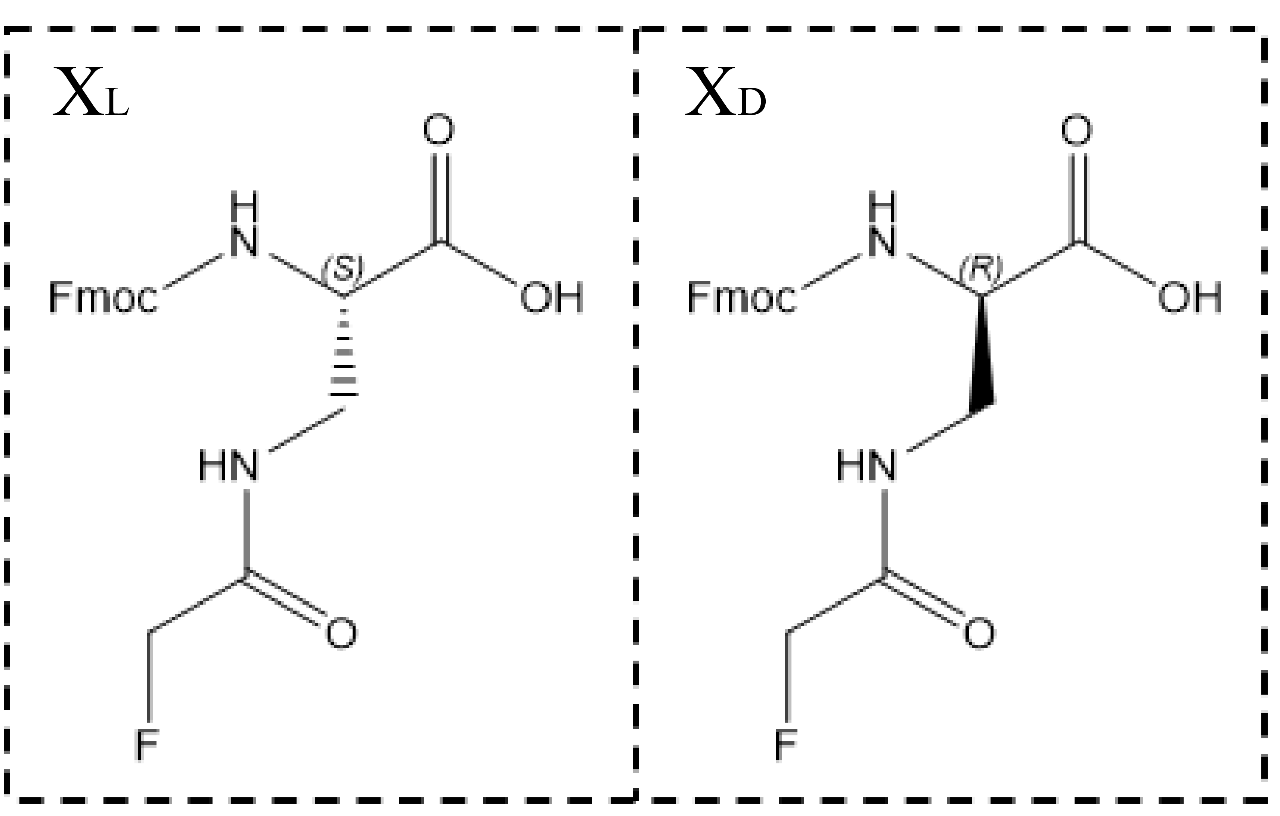
**

**Fig. S1.** The chemical structures of X_L_ and X_D_ as the Fmoc building blocks incorporated into the FTDR peptides, SRC2-LD and SRC2-LL.

**
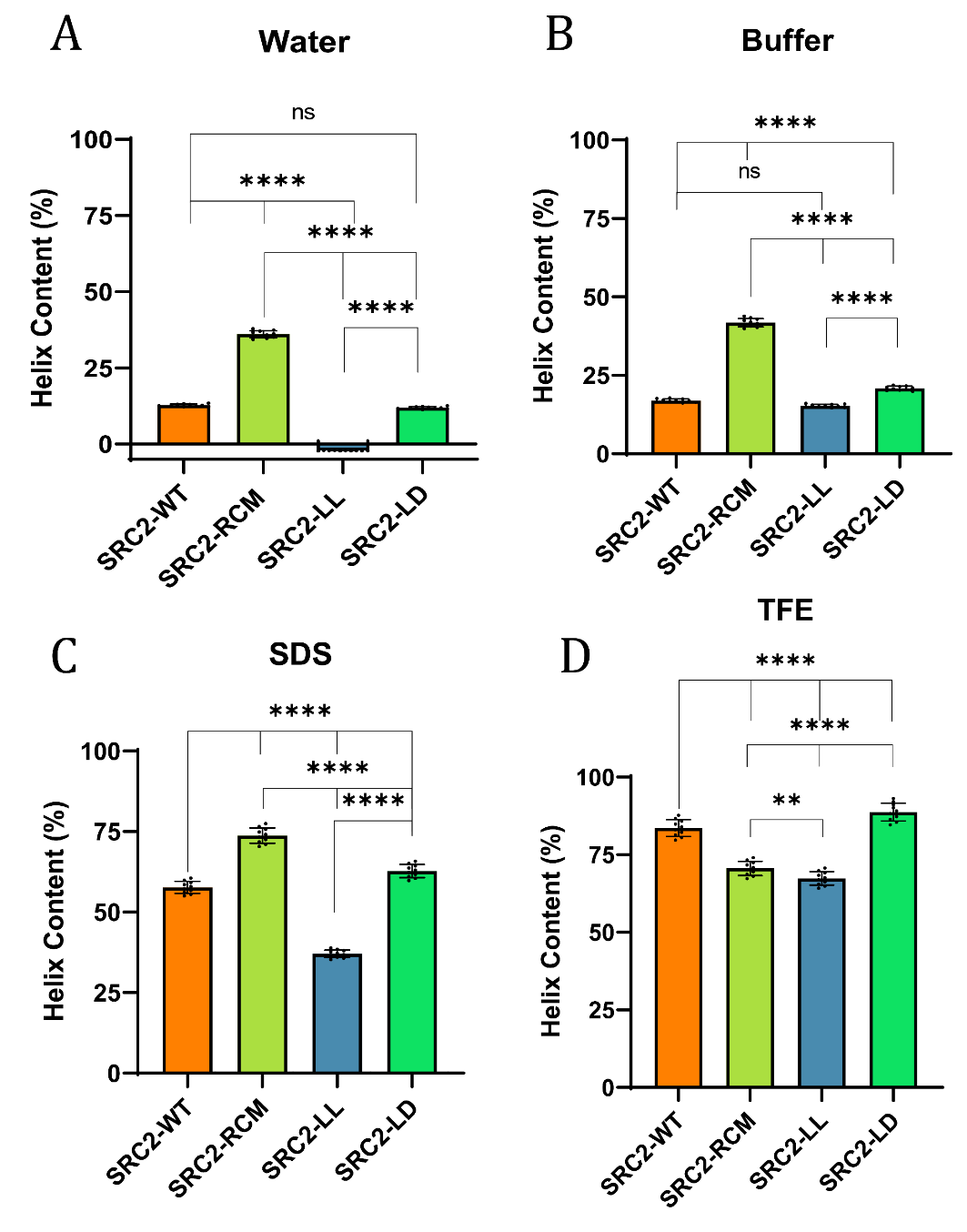
**

**Fig. S2.** Average calculated helicity values (mean ± SD) derived from circular dichroism (CD) measurements of peptides in different solvents. (A) Water, (B) 45 mM phosphate buffer, pH 7.4, (C) 30 mM SDS micellular solution, pH 7.4, and (D) 50% TFE solution, pH 7.4. Each data point represents different helical percentages calculated using variables ranging from 2.5 to 3.4 (n = 10), which are commonly employed in helicity calculations. The initial data represents the average of three accumulated CD scans, given as a single data point by the Jasco software. An ordinary one-way ANOVA was used to assess statistical significance where ‘*’ indicates p < 0.05, ‘**’ indicates p < 0.005, ‘***’ indicates p < 0.0005, and ‘****’ indicates p < 0.0001.


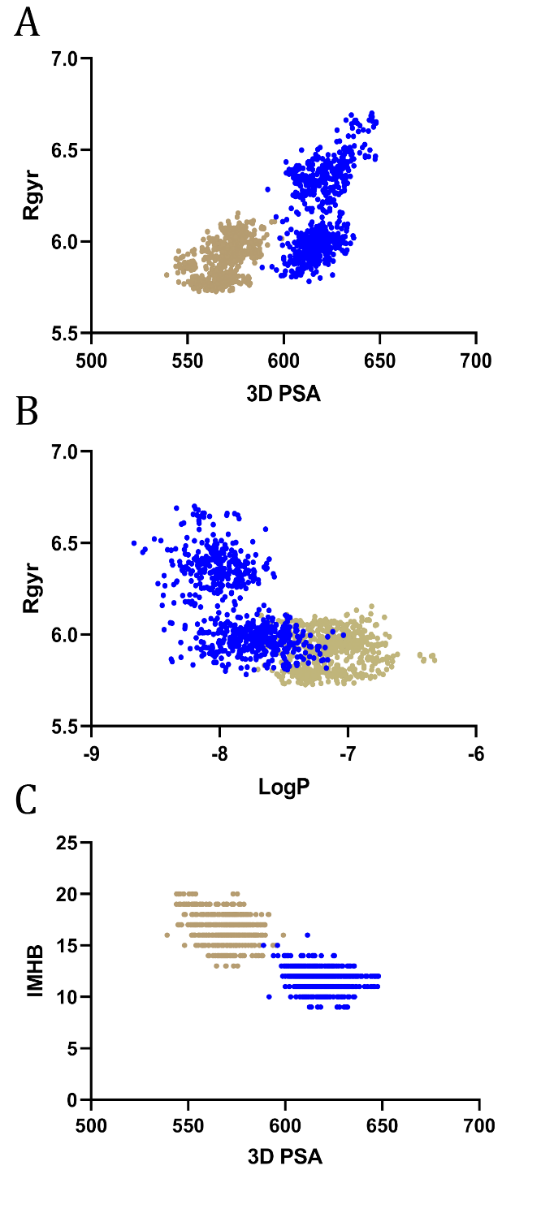


**Fig. S3. Conformational Sampling of SRC2-RCM in water (blue) and chloroform (brown).** (A) 3D polar surface area (PSA) vs radius of gyration (Rgyr). (B) LogP vs Rgyr. (C) 3D-PSA vs number of intramolecular hydrogen bonds (IMHB).


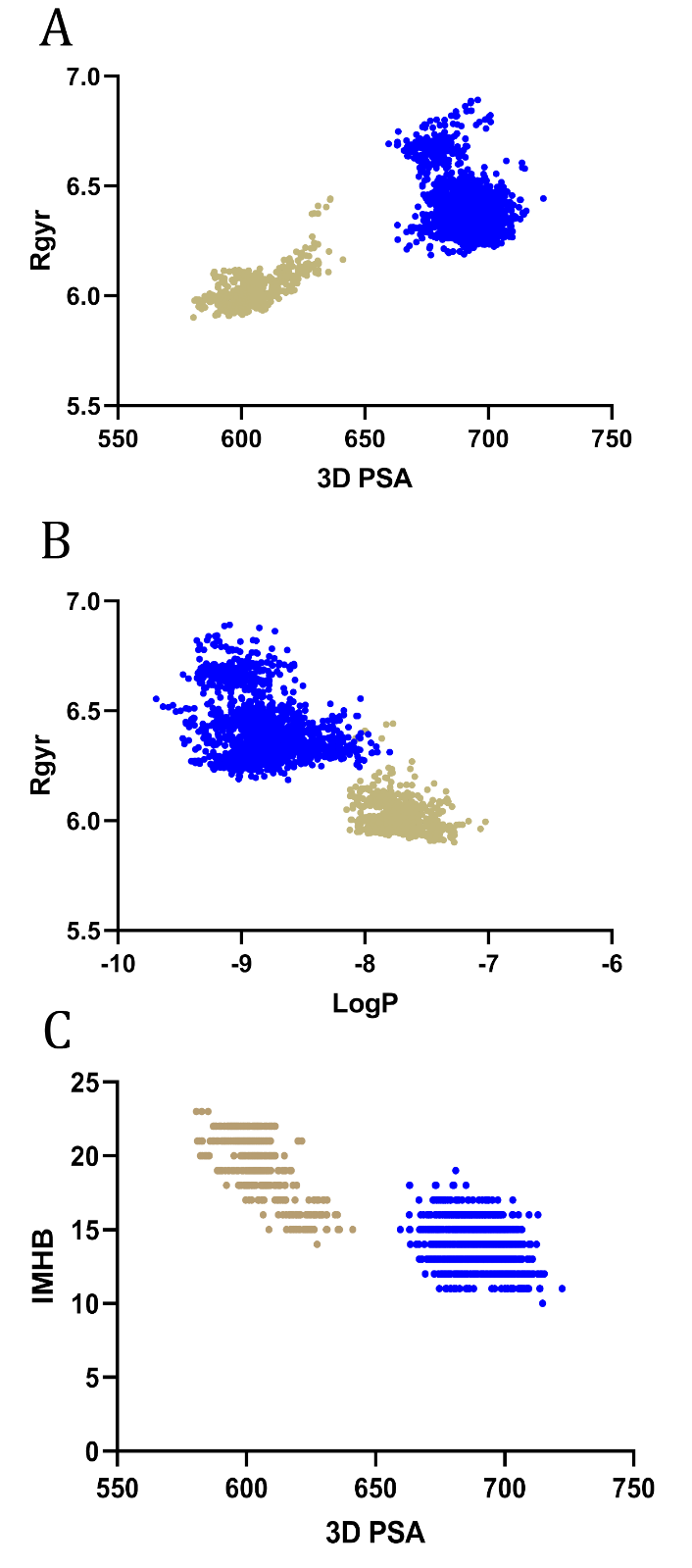


**Fig. S4. Conformational Sampling of SRC2-LD in water (blue) and chloroform (brown).** (A) 3D polar surface area (PSA) vs radius of gyration (Rgyr). (B) LogP vs Rgyr. (C) 3D-PSA vs number of intramolecular hydrogen bonds (IMHB).


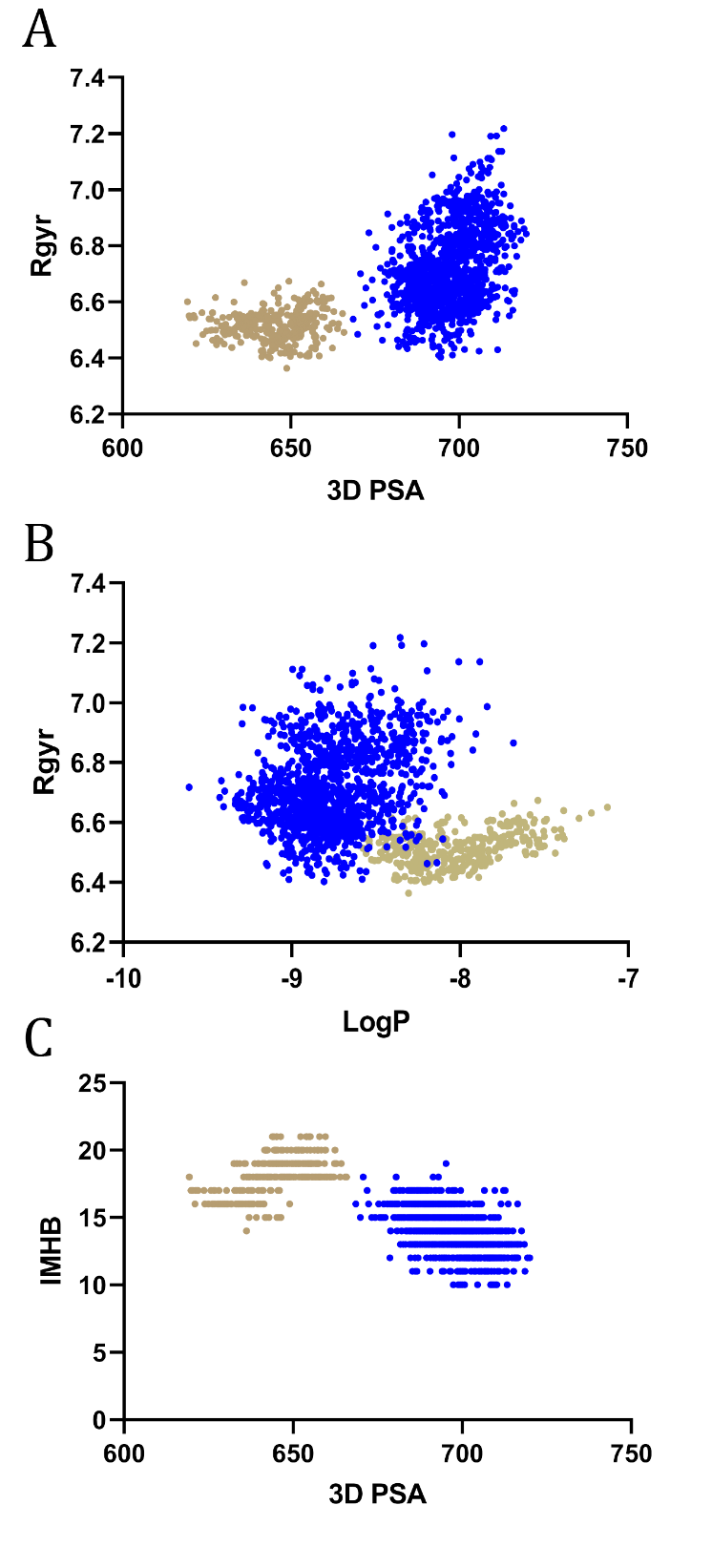


**Fig. S5. Conformational Sampling of SRC2-LL in water (blue) and chloroform (brown).** (A) 3D polar surface area (PSA) vs radius of gyration (Rgyr). (B) LogP vs Rgyr. (C) 3D-PSA vs number of intramolecular hydrogen bonds (IMHB).


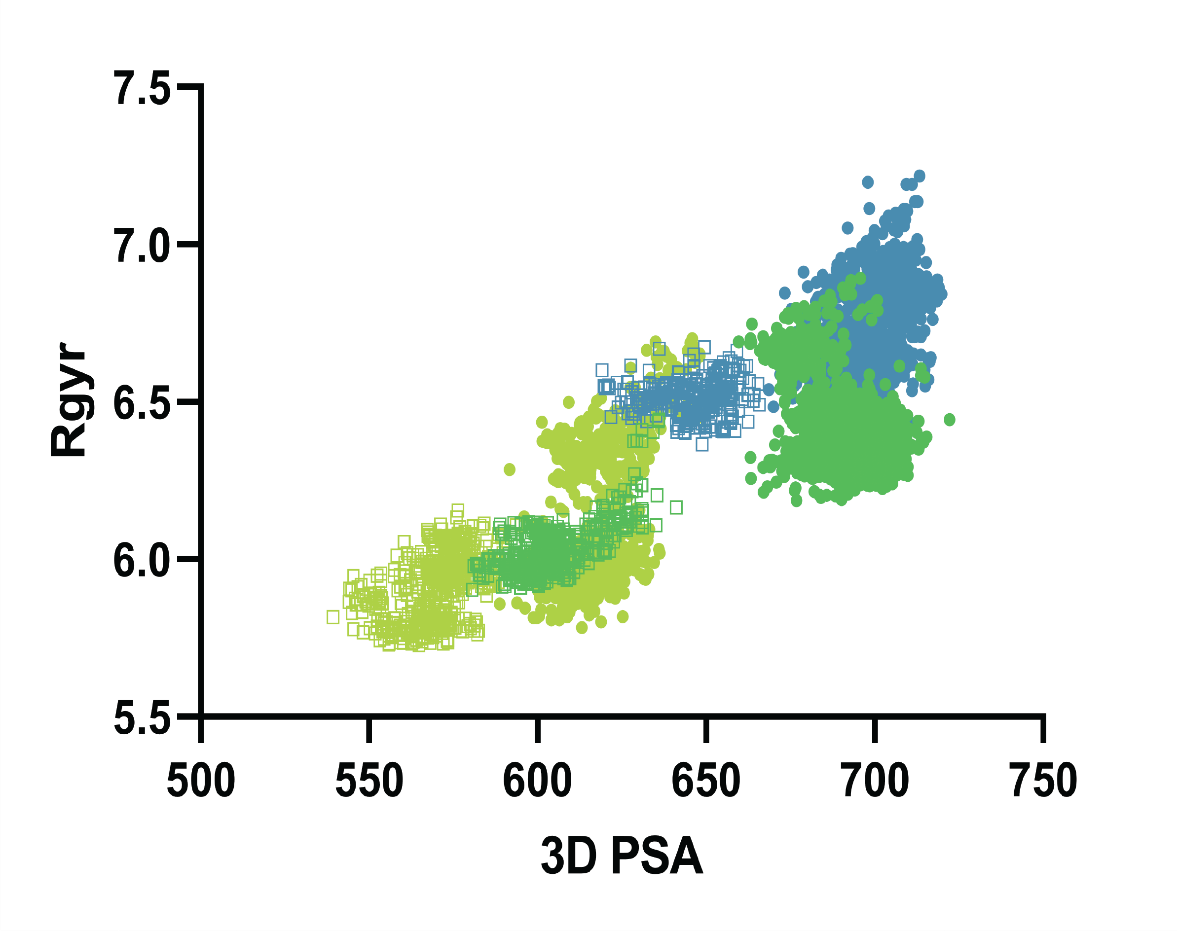

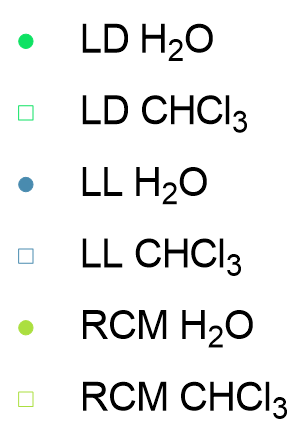


**Fig. S6. 3D-PSA vs Rgyr plot of all sampled conformations** of LD (green), LL (blue), and RCM (yellow) in water (solid circle) and chloroform (hollow square).


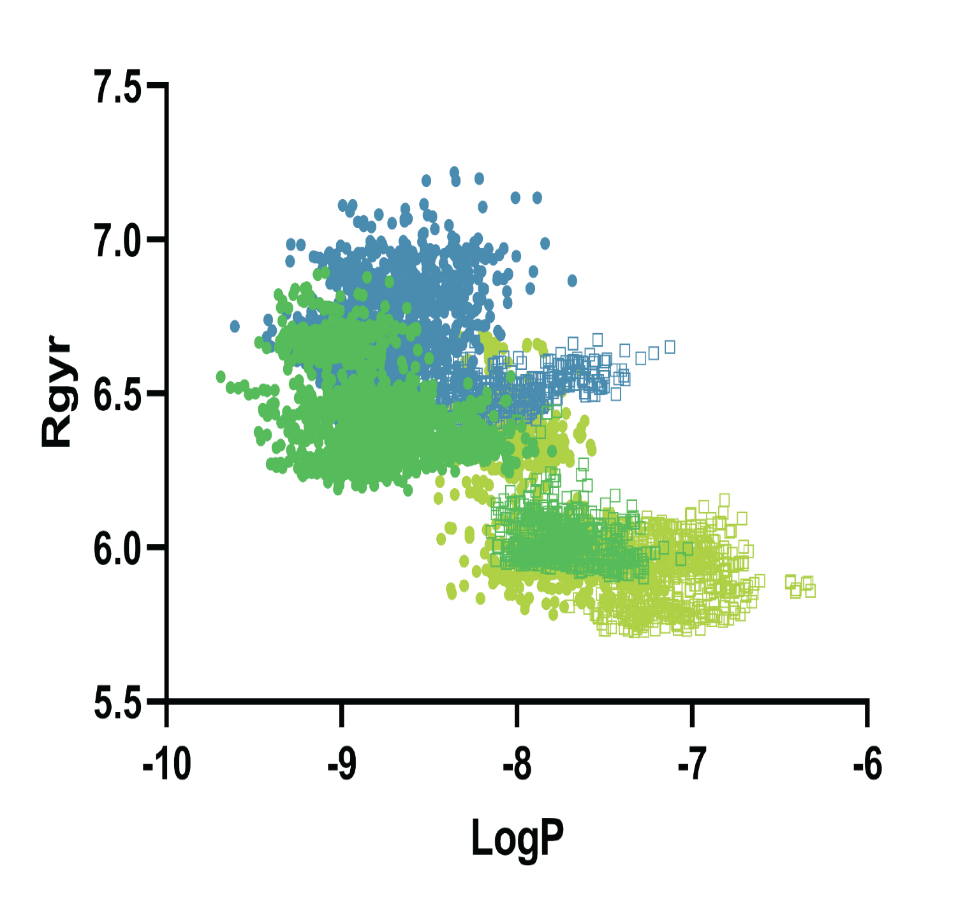

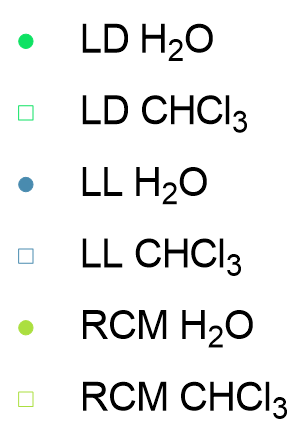


**Fig. S7. LogP vs Rgyr plot of all sampled conformations** of LD (green), LL (blue), and RCM (yellow) in water (solid circle) and chloroform (hollow square).


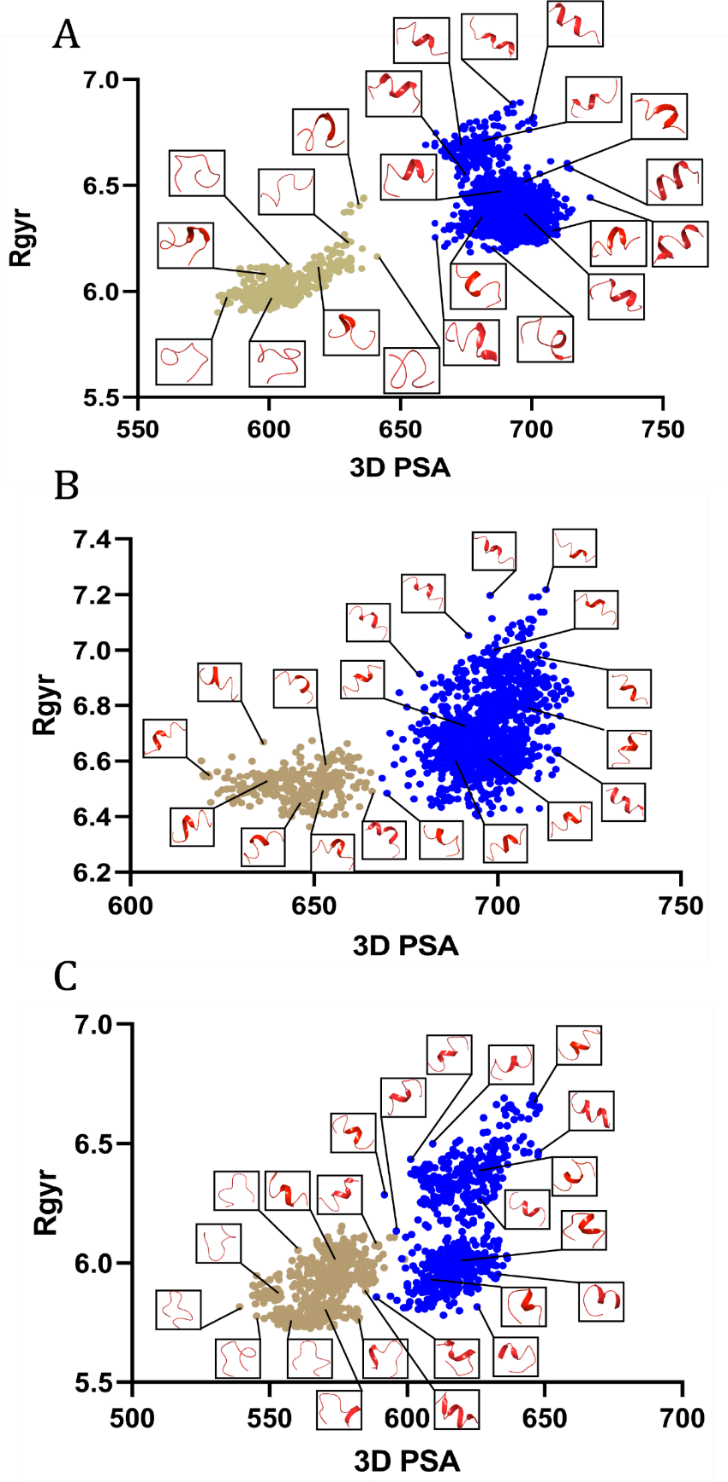


**Fig. S8. An assortment of conformations (inlaid boxes) that illustrate the structural diversity of these peptides in aqueous (water – blue) and membrane-mimicking environments (chloroform – brown).** (A) SRC2-LD, (B) SRC2-LL, and (C) SRC2-RCM.


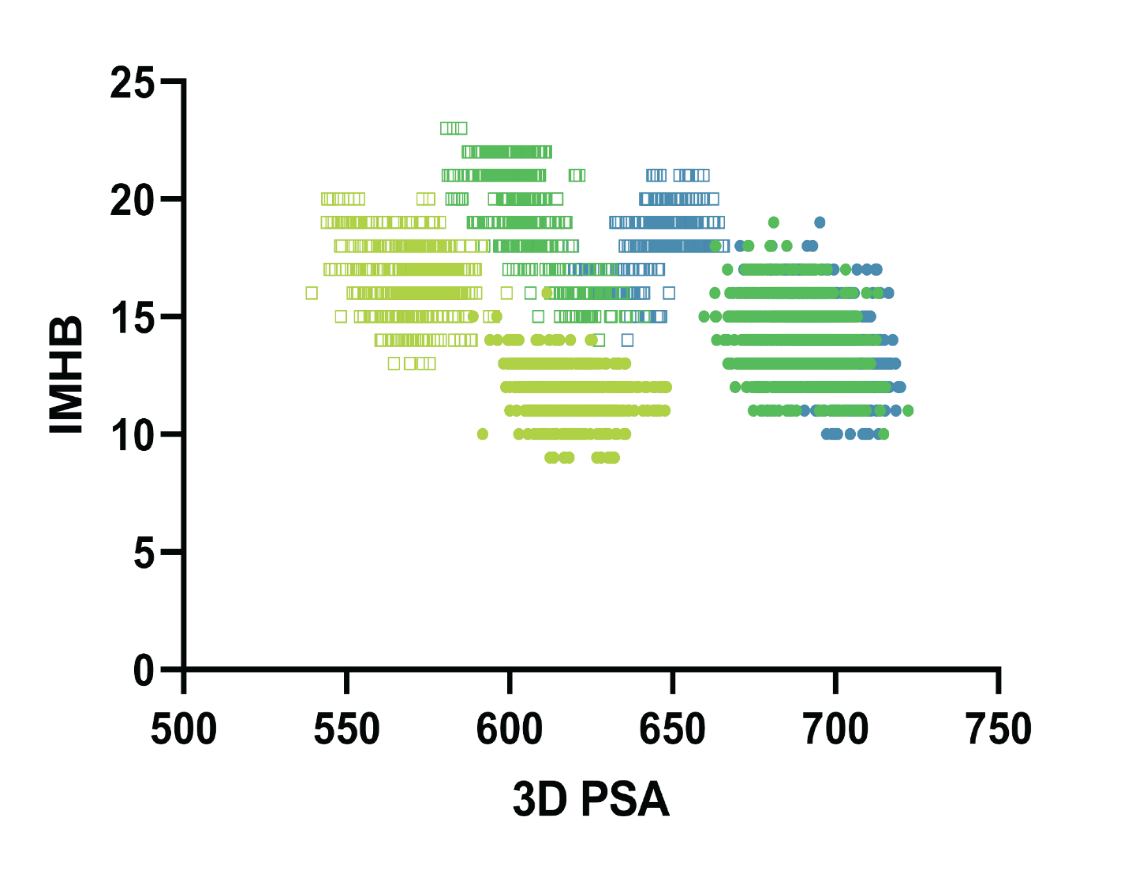

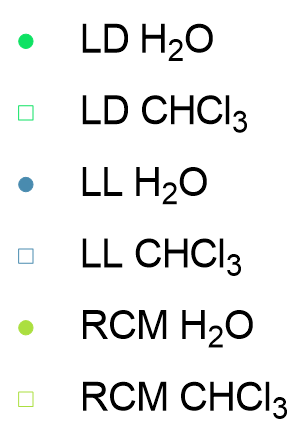


**Fig. S9. 3D-PSA vs number of intramolecular hydrogen bonds (IMHB) plot of all sampled conformations** of LD (green), LL (blue), and RCM (yellow) in water (solid circle) and chloroform (hollow square).


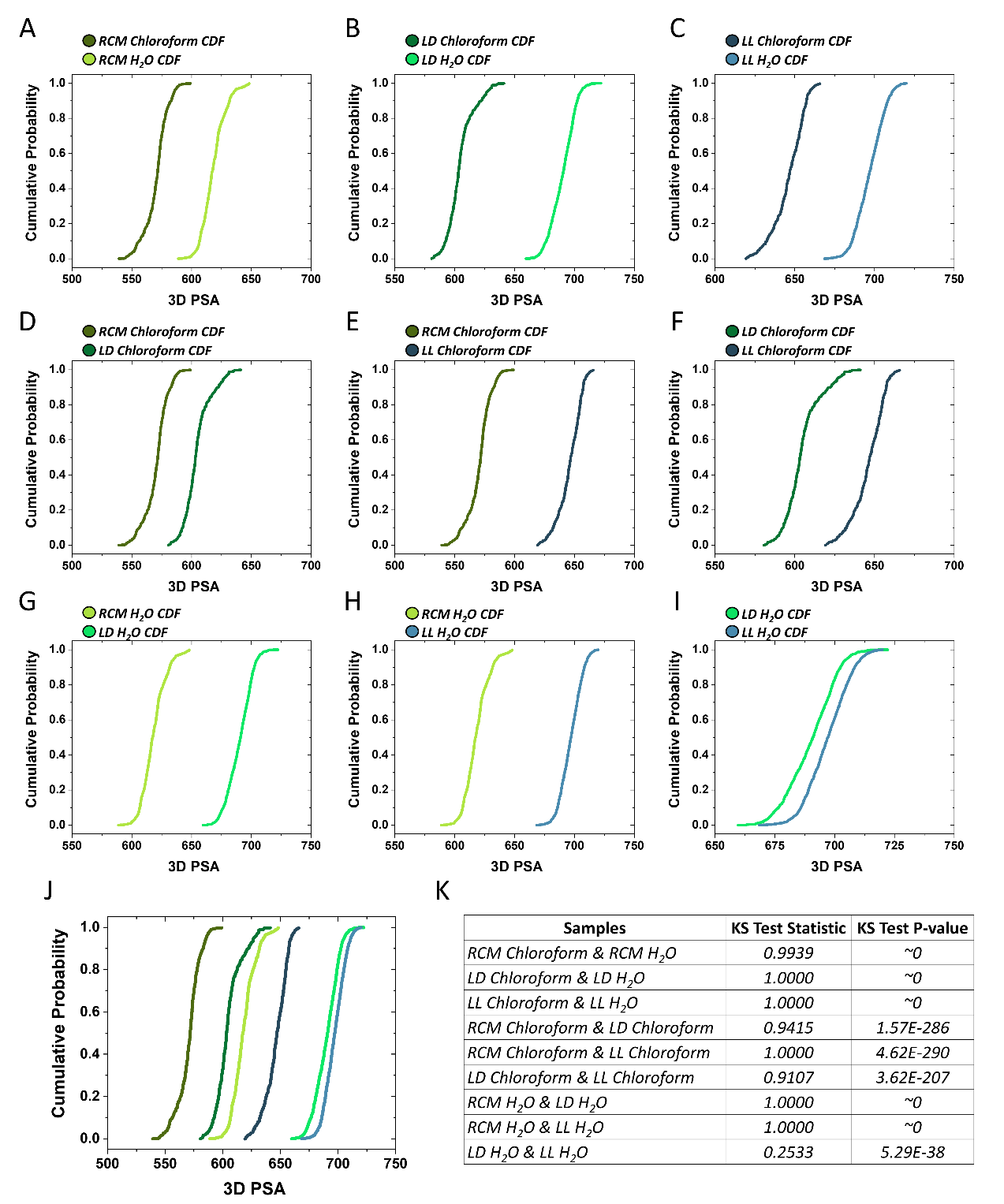


**Fig. S10.** Two-sample Kolmogorov-Smirnov (KS) test of 3D PSA distributions from the in silico modeling results. Shown are the cumulative distribution functions (CDFs) of 3D PSA distributions SRC2-RCM in chloroform (A, D, E), SRC2-RCM in H_2_O (A, G, H), SRC2-LD in chloroform (B, D, F), SRC2-LD in H_2_O (B, G, I), SRC2-LL in chloroform (C, E, F), and SRC2-LL in H_2_O (C, H, I). (J) Superimposed CDFs from A – I. (K) Table of the KS test statistic, which is the maximum cumulative probability distance between any two CDFs and the KS test p-value.


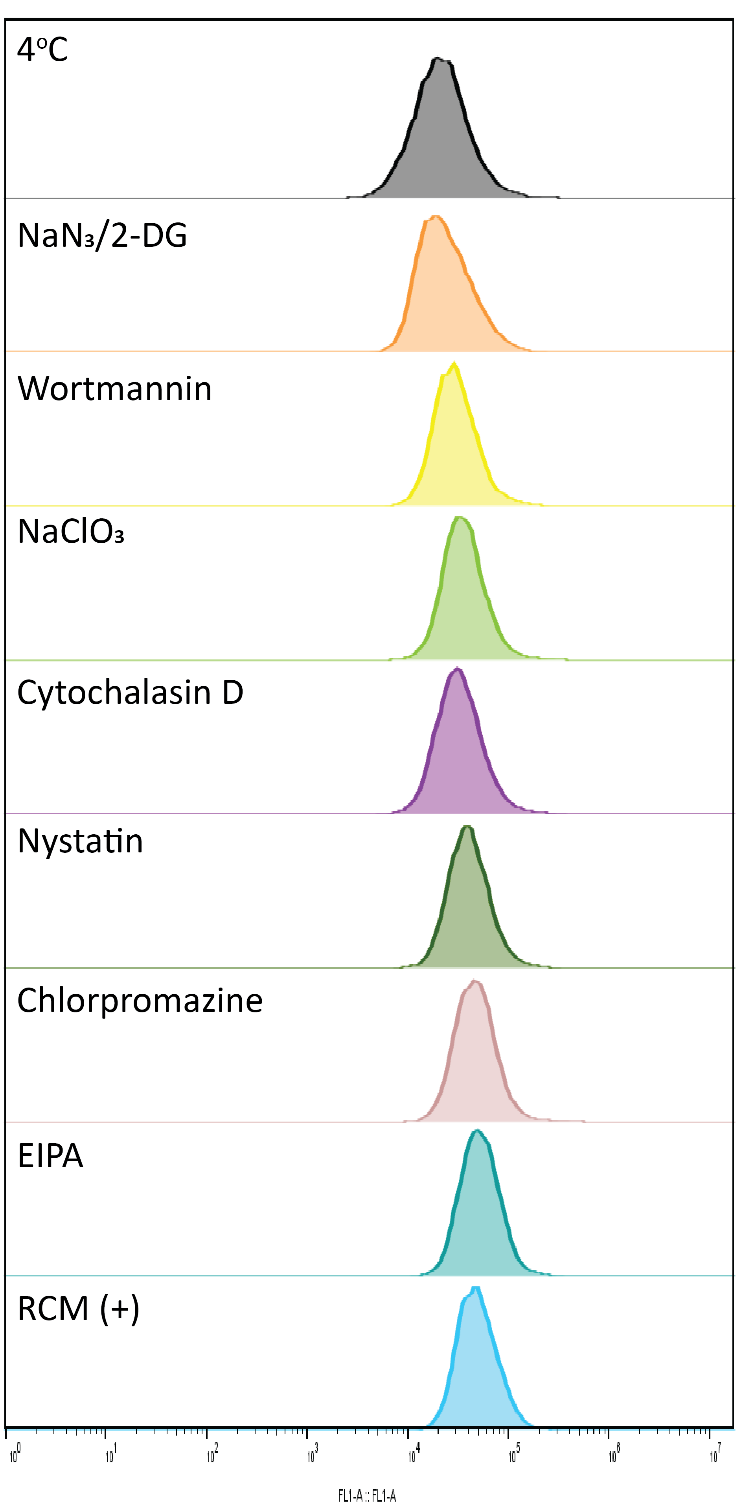


**Fig. S11. Representative histograms for SRC2-RCM cellular uptake assay.** ‘RCM (+)’ represents the uninhibited positive control, while the other panels denote various blocker treatments.


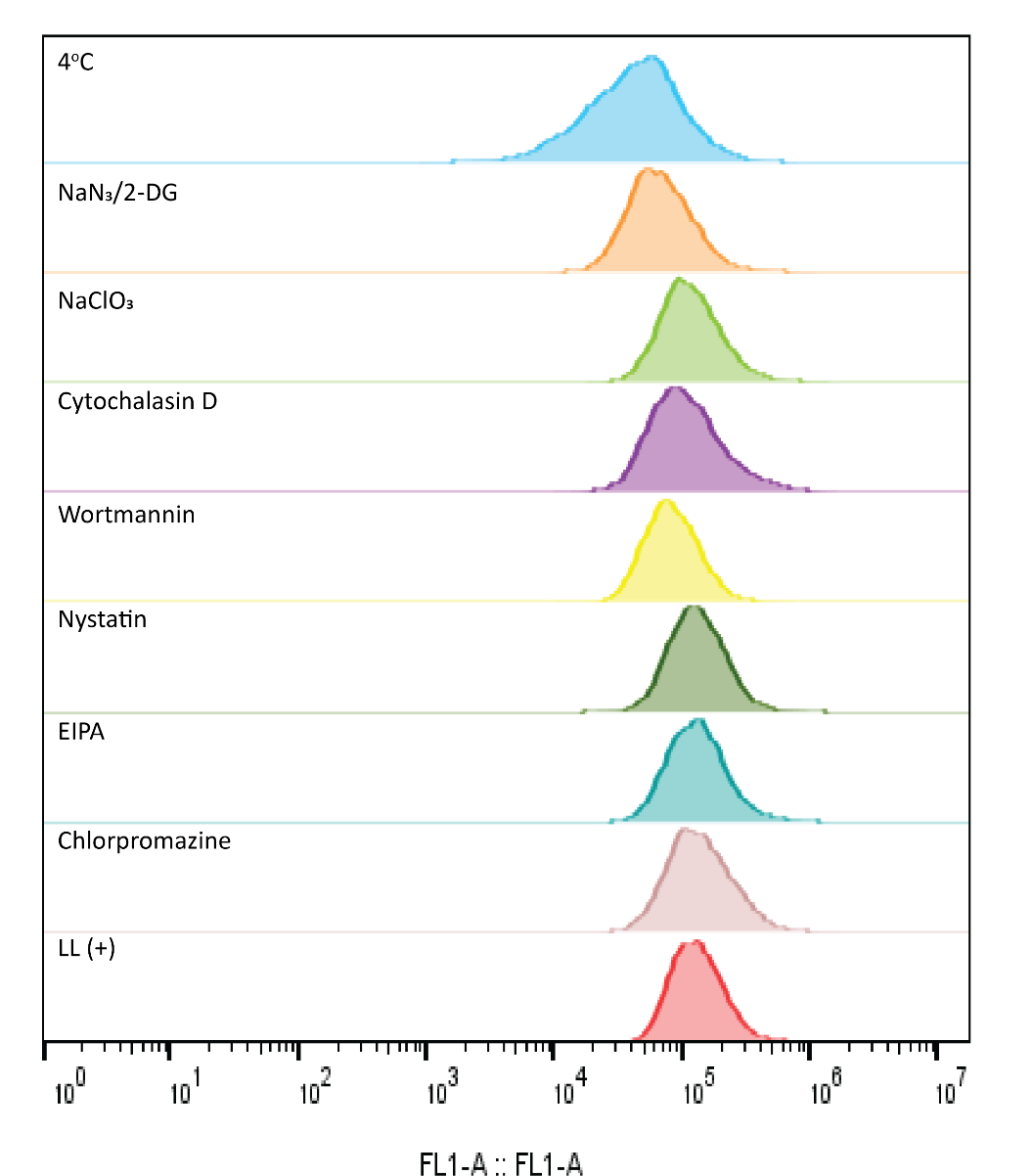


**Fig. S12. Representative histograms for SRC2-LL cellular uptake assay.** ‘LL (+)’ represents the uninhibited positive control, while the other panels denote various blocker treatments.


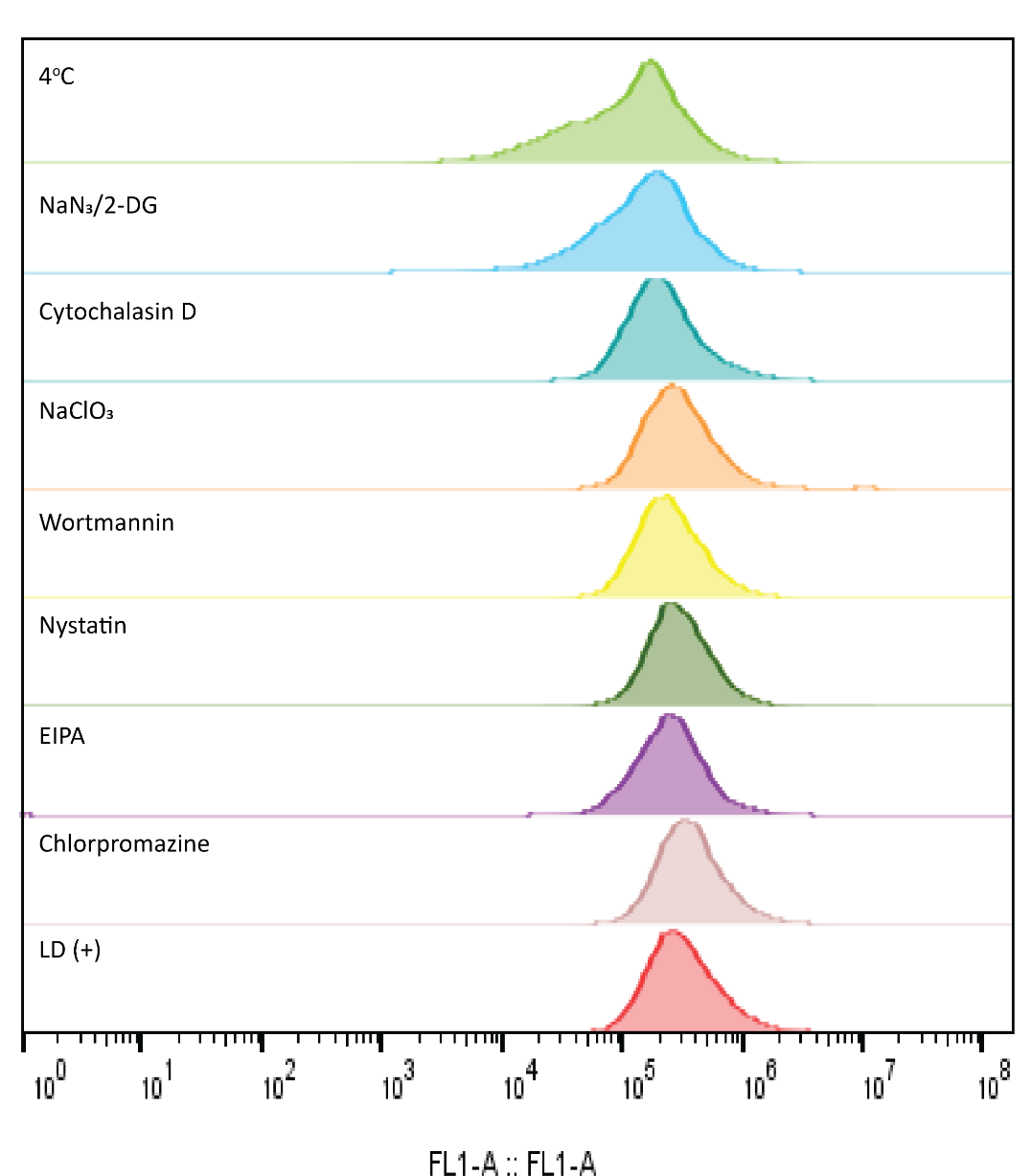


**Fig. S13. Representative histograms for SRC2-LD cellular uptake assay.** ‘LD (+)’ represents the uninhibited positive control, while the other panels denote various blocker treatments.


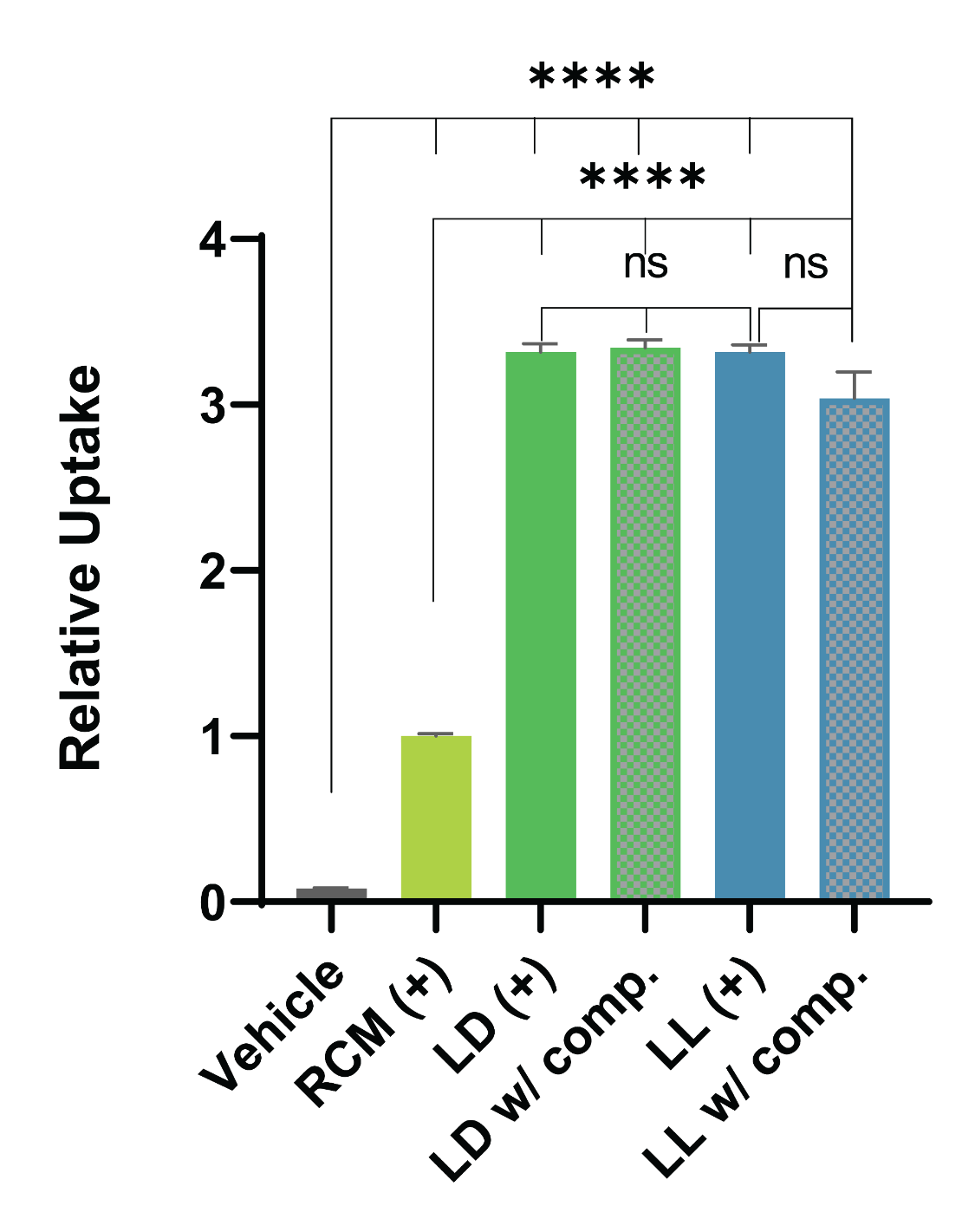


**Fig. S14. Competition cellular uptake assay.** MCF-7 cells were incubated with 15 μM of FITC-labeled peptides with or without a 30-minute pretreatment of 100-fold excess of unlabeled peptide (1.5 mM) and incubated for 24 hours. Data represent triplicate wells (Mean ± SD) normalized to relative uptake of SRC2-RCM. A minimum of 10,000 single cells (n ≥ 10,000) were analyzed for each group. Statistical significance was determined using an unpaired two-sided Welch’s t test, where ‘****’ indicates a p-value < 0.0001.


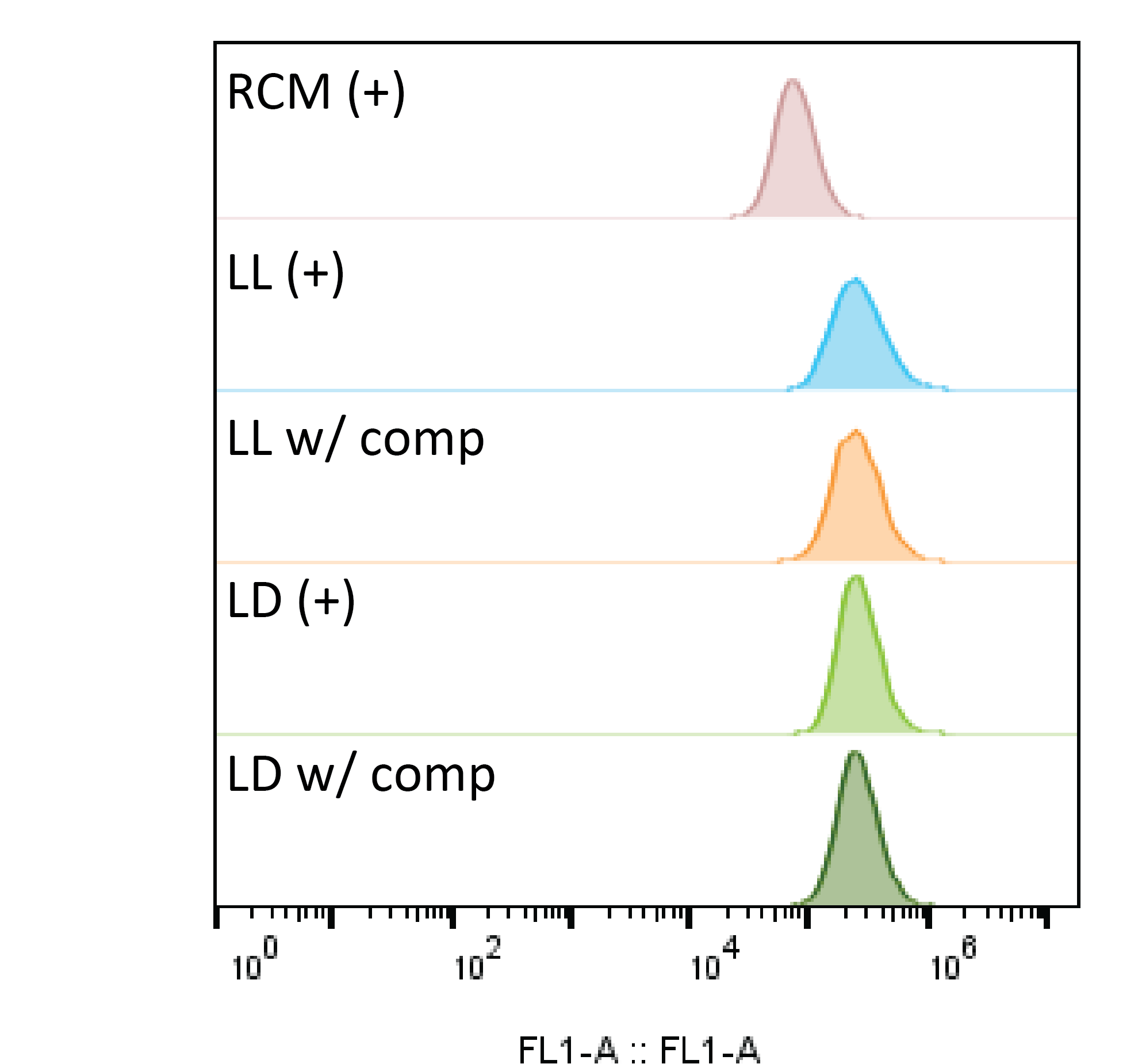


**Fig. S15. Representative histograms for the competition cellular uptake assay.** ‘(+)’ represents the positive control (only FITC-peptide), while ‘w/ comp’ denotes the presence of a 100-fold excess unlabeled peptide.

**Fig. S16. MCF-7 cytotoxicity assay of endocytic blockers used in flow cytometry uptake experiments.** Cells were incubated with blockers for a total of 5 hrs, mirroring the time used for the flow cytometry cell uptake experiments in the main text. Data was measured in duplicates from three independent experiments by Cell Titer Glo luminescence. Data shown as mean ± SD and was normalized to vehicle (n = 6). To assess statistical significance a Welch’s unpaired two-tailed t test was applied for each group compared to the vehicle. ‘**’ indicates a p-value < 0.005, ‘***’ indicates a p-value < 0.0005, and ‘****’ indicates a p-value < 0.0001.


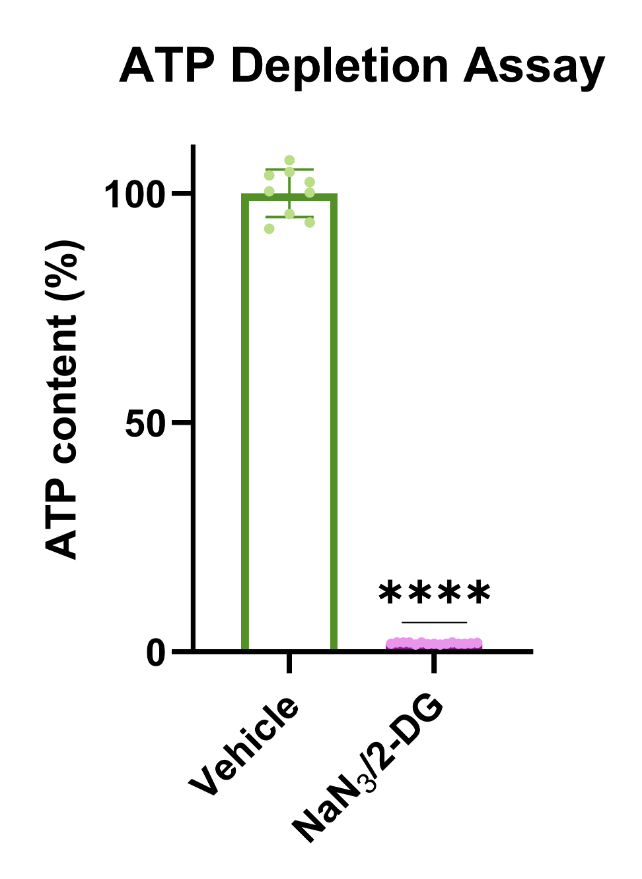


**Fig. S17. ATP depletion assay in MCF-7 cells.** Total treatment time of 5 h to match the incubation time used in the endocytic blocker assay. Final concentrations for NaN_3_ and 2-deoxy-D-glucose (2-DG) were 10 mM and 30 mM, respectively. Data was measured in triplicates from three independent experiments by Cell Titer Glo luminescence and was normalized to vehicle (n = 9). To assess statistical significance a Welch’s unpaired two-tailed t test was applied for each group compared to the vehicle where ‘****’ indicates a p-value < 0.0001.

**Fig. S18. Quantification of Co-IP assays.** Data represents three independent experiments for the WT and E2 + WT groups, and four independent experiments for all other groups. Statistical significance was determined using an ordinary one-way ANOVA where ‘*’ indicates p < 0.05 and ‘**’ indicates p < 0.01.


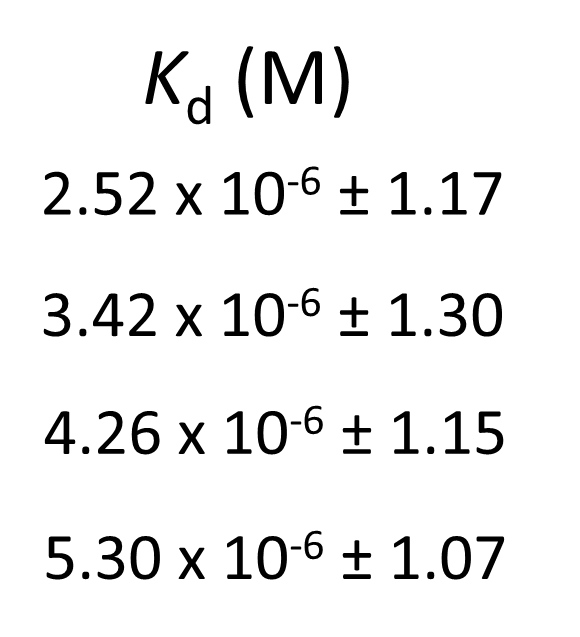


**Fig. S19. Fluorescence anisotropy assay of FITC-peptides’ binding affinity to the ERα ligand-binding domain.** Experiments were performed in duplicate and were replicated independently (n = 4). Kd was extrapolated by GraphPad Prism (version. 8) using a sigmoidal concentration-response curve. Data was represented as mean ± standard error.


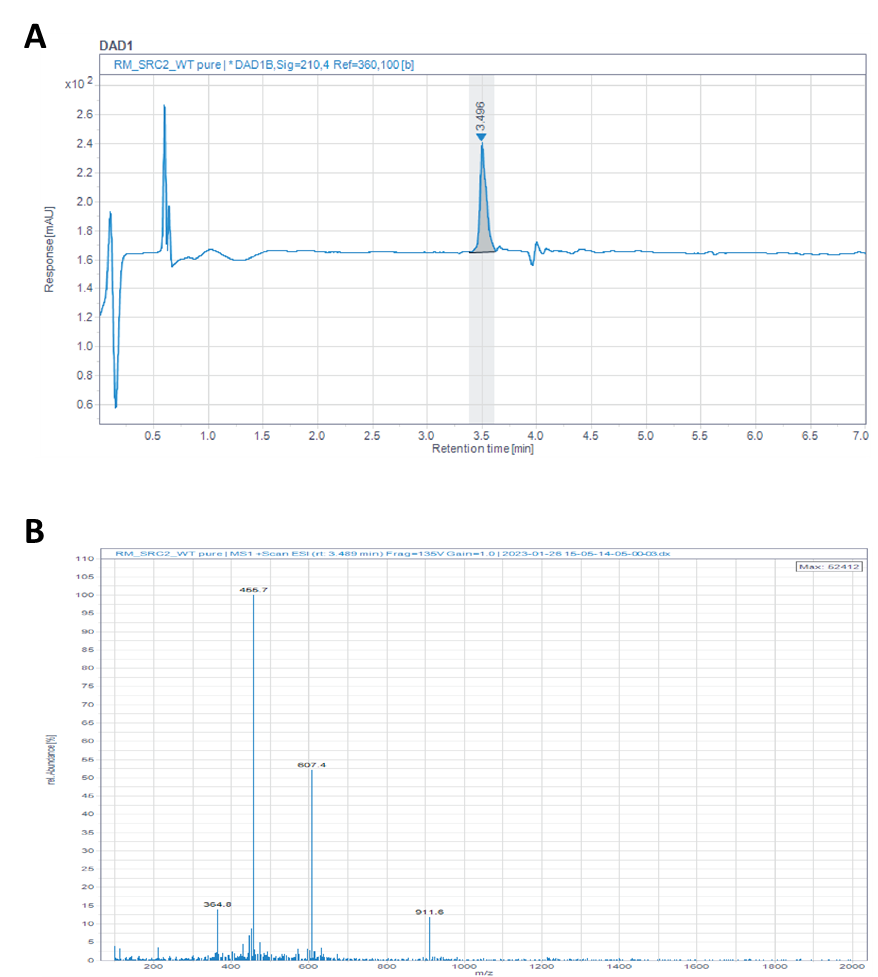


**Fig. S20. LC-MS spectra of synthesized and purified SRC2-WT peptide.** (A) The desired peptide peak retention time is 3.496 min. (B) The expected peptide mass is 1819.08 Da. In the ESI-MS window, 911.6 Da is observed for [M+2H]/2; 607.4 Da is observed for [M+3H]/3; 455.7 Da is observed for [M+4H]/4; and 364.8 Da is observed for [M+5H]/5.


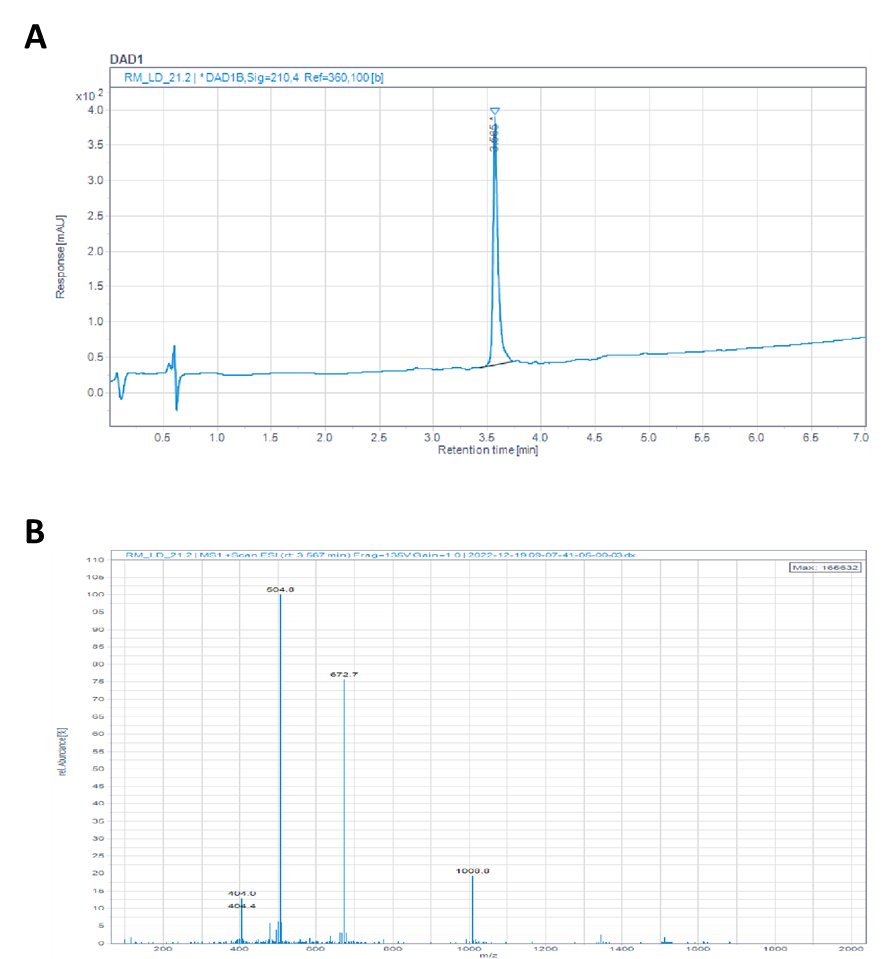


## **Fig. S21. LC-MS spectra of synthesized and purified SRC2-LD peptide.** (A) The desired peptide peak retention time is 3.565 min. (B) The expected peptide mass is 2015.27Da. In the ESI-MS window, 1008.8 Da is observed for [M+2H]/2; 672.7 Da is observed for [M+3H]/3; 504.8 Da is observed for [M+4H]/4; and 404.0 Da is observed for [M+5H]/5.


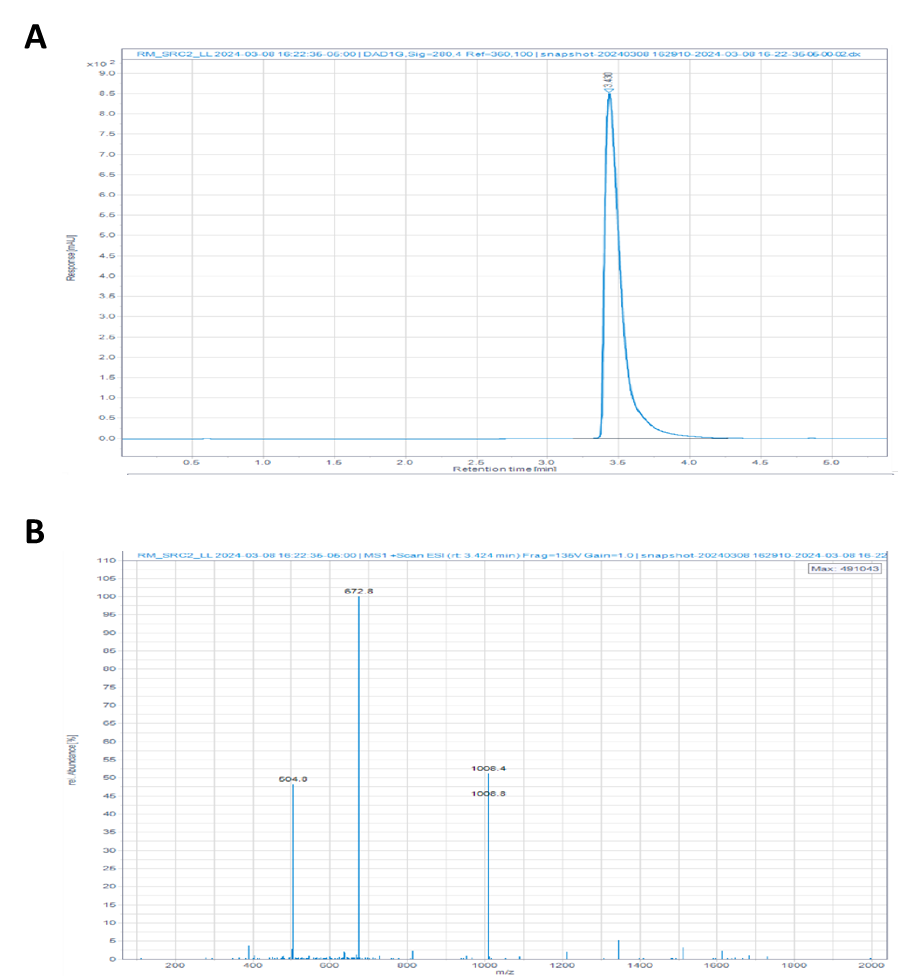


**Fig. S22. LC-MS spectra of synthesized and purified SRC2-LL peptide.** (A) The desired peptide peak retention time is 3.430 min. (B) The expected peptide mass is 2015.27 Da. In the ESI-MS window, 1008.8 Da is observed for [M+2H]/2; 672.8 Da is observed for [M+3H]/3; and 504.8 Da is observed for [M+4H]/4.


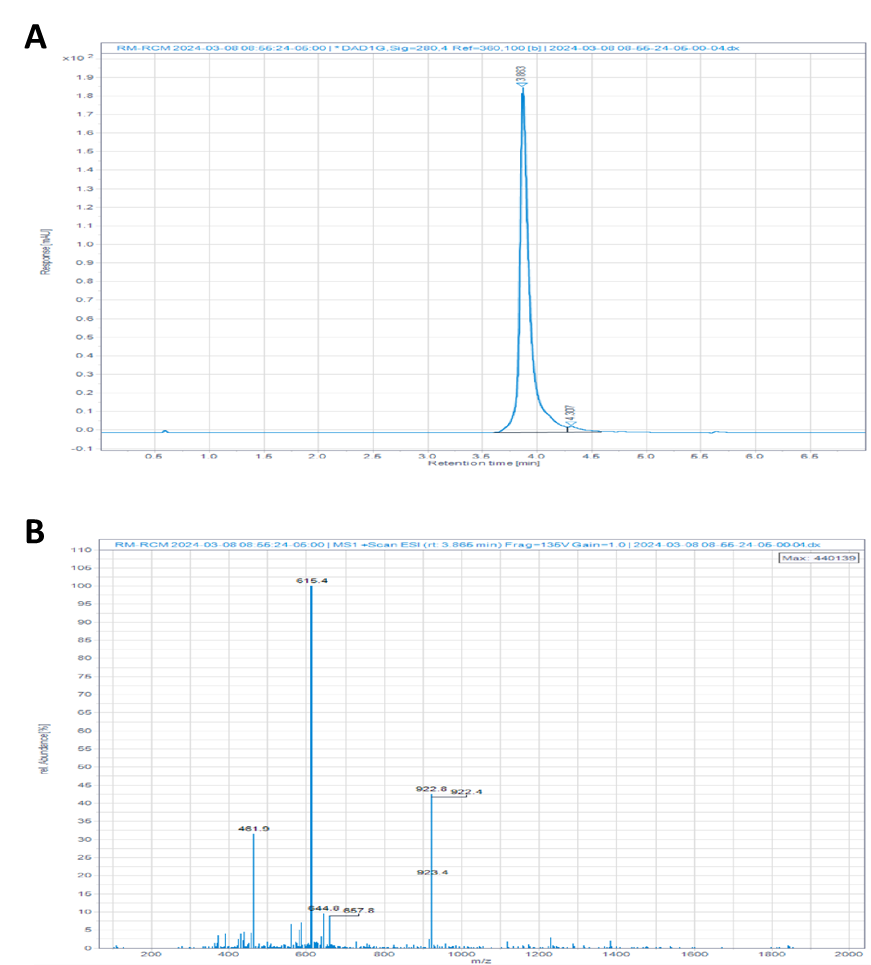


**Fig. S23. LC-MS spectra of synthesized and purified SRC2-RCM**. (A) The desired peptide peak retention time is 3.863 min. (B) The expected peptide mass is 1843.10 Da. In the ESI-MS window, 922.8 Da is observed for [M+2H]/2; 615.4 Da is observed for [M+3H]/3; and 461.9 Da is observed for [M+4H]/4.
